# Optogenetic tachypacing facilitates burst-induced arrhythmia in stem cell-derived human atrial engineered heart tissue

**DOI:** 10.1093/europace/euag158

**Published:** 2026-08-18

**Authors:** Karl-Felix Müller, Bangfen Pan, Julius Ridder, Jessica Schrapers, Julius Hansen, Tandy M Meier, Carl Schulz, Tim Stüdemann, Marta Lemme, Julia Krause, Marc N Hirt, Aaltje Maria Stella Stoter, Ingke Braren, Michael Spohn, Florian Weinberger, Larissa Fabritz, Xiaodong Duan, Shiqiang Gao, Christine E Gee, Arne Hansen, Thomas Eschenhagen, Torsten Christ, Justus Stenzig

**Affiliations:** Department of Experimental Pharmacology and Toxicology, University Medical Center Hamburg-Eppendorf, Martinistrasse 52, 20246 Hamburg, Germany; DZHK (German Center for Cardiovascular Research), Partner Site Hamburg/Kiel/Lübeck, 20246 Hamburg, Germany; Department of Cardiology, University Medical Center Hamburg-Eppendorf, 20246 Hamburg, Germany; Department of Experimental Pharmacology and Toxicology, University Medical Center Hamburg-Eppendorf, Martinistrasse 52, 20246 Hamburg, Germany; DZHK (German Center for Cardiovascular Research), Partner Site Hamburg/Kiel/Lübeck, 20246 Hamburg, Germany; Institute of Experimental Cardiology, University Hospital Heidelberg, 69120 Heidelberg, Germany; DZHK (German Center for Cardiovascular Research), Partner Site Hamburg/Kiel/Lübeck, 20246 Hamburg, Germany; Department of Cardiology, University Medical Center Hamburg-Eppendorf, 20246 Hamburg, Germany; University Center of Cardiovascular Science, University Heart and Vascular Center Hamburg, 20246 Hamburg, Germany; Department of Experimental Pharmacology and Toxicology, University Medical Center Hamburg-Eppendorf, Martinistrasse 52, 20246 Hamburg, Germany; DZHK (German Center for Cardiovascular Research), Partner Site Hamburg/Kiel/Lübeck, 20246 Hamburg, Germany; Sarcura GmbH, 3400 Klosterneuburg, Austria; Department of Experimental Pharmacology and Toxicology, University Medical Center Hamburg-Eppendorf, Martinistrasse 52, 20246 Hamburg, Germany; DZHK (German Center for Cardiovascular Research), Partner Site Hamburg/Kiel/Lübeck, 20246 Hamburg, Germany; Department of Experimental Pharmacology and Toxicology, University Medical Center Hamburg-Eppendorf, Martinistrasse 52, 20246 Hamburg, Germany; DZHK (German Center for Cardiovascular Research), Partner Site Hamburg/Kiel/Lübeck, 20246 Hamburg, Germany; Department of Cardiology, University Medical Center Hamburg-Eppendorf, 20246 Hamburg, Germany; Department of Experimental Pharmacology and Toxicology, University Medical Center Hamburg-Eppendorf, Martinistrasse 52, 20246 Hamburg, Germany; DZHK (German Center for Cardiovascular Research), Partner Site Hamburg/Kiel/Lübeck, 20246 Hamburg, Germany; Department of Experimental Pharmacology and Toxicology, University Medical Center Hamburg-Eppendorf, Martinistrasse 52, 20246 Hamburg, Germany; Nanion Technologies GmbH, 80339 Munich, Germany; DZHK (German Center for Cardiovascular Research), Partner Site Hamburg/Kiel/Lübeck, 20246 Hamburg, Germany; Department of Cardiology, University Medical Center Hamburg-Eppendorf, 20246 Hamburg, Germany; Department of Experimental Pharmacology and Toxicology, University Medical Center Hamburg-Eppendorf, Martinistrasse 52, 20246 Hamburg, Germany; DZHK (German Center for Cardiovascular Research), Partner Site Hamburg/Kiel/Lübeck, 20246 Hamburg, Germany; University Center of Cardiovascular Science, University Heart and Vascular Center Hamburg, 20246 Hamburg, Germany; Department of Experimental Pharmacology and Toxicology, University Medical Center Hamburg-Eppendorf, Martinistrasse 52, 20246 Hamburg, Germany; DZHK (German Center for Cardiovascular Research), Partner Site Hamburg/Kiel/Lübeck, 20246 Hamburg, Germany; Vector Facility, University Medical Center Hamburg-Eppendorf, 20246 Hamburg, Germany; Bioinformatics Core Facility, University Medical Center Hamburg-Eppendorf, 20246 Hamburg, Germany; Department of Experimental Pharmacology and Toxicology, University Medical Center Hamburg-Eppendorf, Martinistrasse 52, 20246 Hamburg, Germany; DZHK (German Center for Cardiovascular Research), Partner Site Hamburg/Kiel/Lübeck, 20246 Hamburg, Germany; Research Group Tissue Engineering and Regenerative Therapies, Centro Nacional de Investigaciones Cardiovasculares Carlos III (CNIC), Madrid, Spain; DZHK (German Center for Cardiovascular Research), Partner Site Hamburg/Kiel/Lübeck, 20246 Hamburg, Germany; Department of Cardiology, University Medical Center Hamburg-Eppendorf, 20246 Hamburg, Germany; University Center of Cardiovascular Science, University Heart and Vascular Center Hamburg, 20246 Hamburg, Germany; Department of Neurophysiology, Institute of Physiology, University of Würzburg, Würzburg, Germany; Department of Neuroscience, School of Life Sciences, Southern University of Science and Technology, Shenzhen 518055, China; Department of Neurophysiology, Institute of Physiology, University of Würzburg, Würzburg, Germany; Department of Synaptic Neuroscience, Center for Molecular Neurobiology (ZMNH), University Medical Center Hamburg-Eppendorf, 20251 Hamburg, Germany; Department of Experimental Pharmacology and Toxicology, University Medical Center Hamburg-Eppendorf, Martinistrasse 52, 20246 Hamburg, Germany; DZHK (German Center for Cardiovascular Research), Partner Site Hamburg/Kiel/Lübeck, 20246 Hamburg, Germany; Department of Experimental Pharmacology and Toxicology, University Medical Center Hamburg-Eppendorf, Martinistrasse 52, 20246 Hamburg, Germany; DZHK (German Center for Cardiovascular Research), Partner Site Hamburg/Kiel/Lübeck, 20246 Hamburg, Germany; Department of Experimental Pharmacology and Toxicology, University Medical Center Hamburg-Eppendorf, Martinistrasse 52, 20246 Hamburg, Germany; DZHK (German Center for Cardiovascular Research), Partner Site Hamburg/Kiel/Lübeck, 20246 Hamburg, Germany; Department of Experimental Pharmacology and Toxicology, University Medical Center Hamburg-Eppendorf, Martinistrasse 52, 20246 Hamburg, Germany; DZHK (German Center for Cardiovascular Research), Partner Site Hamburg/Kiel/Lübeck, 20246 Hamburg, Germany

**Keywords:** Optogenetics, Tissue engineering, iPS cells, Atrial fibrillation

## Abstract

**Aims:**

Large animal models for atrial fibrillation (AF) research are ethically challenging and costly. Suitable humanized *in vitro* models could circumvent these problems. Here, we aimed to combine tissue engineering, human induced pluripotent stem cell-derived atrial cardiomyocytes (hiPSC-aCM), and optogenetic pacing to trigger an *in vitro* fibrillation-like state, which can be analysed by simple video recording.

**Methods and results:**

Atrial engineered heart tissue (aEHT) was created from hiPSC-aCM and transduced with adeno-associated virus vectors to express the channelrhodopsins CheRiff2.0, Chronos, or PsCatCh2.0f, respectively. We developed novel optogenetic hardware, based on microcontroller programmable light-emitting diodes. The interplay between aEHTs and hardware was optimized, and different pacing patterns were evaluated. Electrical burst pacing was evaluated for arrhythmia induction. Tissue constructs were analysed with regard to action potential, gene and protein expression, and structure. We found our optimized CheRiff2.0 best suitable for optogenetic pacing of aEHT at up to 5 Hz. Atrial EHT could be faithfully tachypaced at 4 Hz for 2.5 weeks. After 5 weeks, electrical burst pacing (20 Hz, 0.5 s) successfully induced self-sustained episodes of a state of fast beating, lower force, and incomplete relaxation. The propensity of these burst-evoked episodes of fibrillation was 3.2-fold higher in long-term paced aEHT than in non-paced controls. Action potential shape and gene expression recapitulated typical features of AF in the tachypaced aEHTs.

**Conclusion:**

We successfully created a first-of-its-kind *in vitro* model of AF, recapitulating the self-sustainability of atrial arrhythmia (‘AF begets AF’). Fibrillation-like episodes can be verified by visual inspection, rendering analysis possible without an electrophysiological setup.

## Graphical Abstract

**Graphical Abstract:**
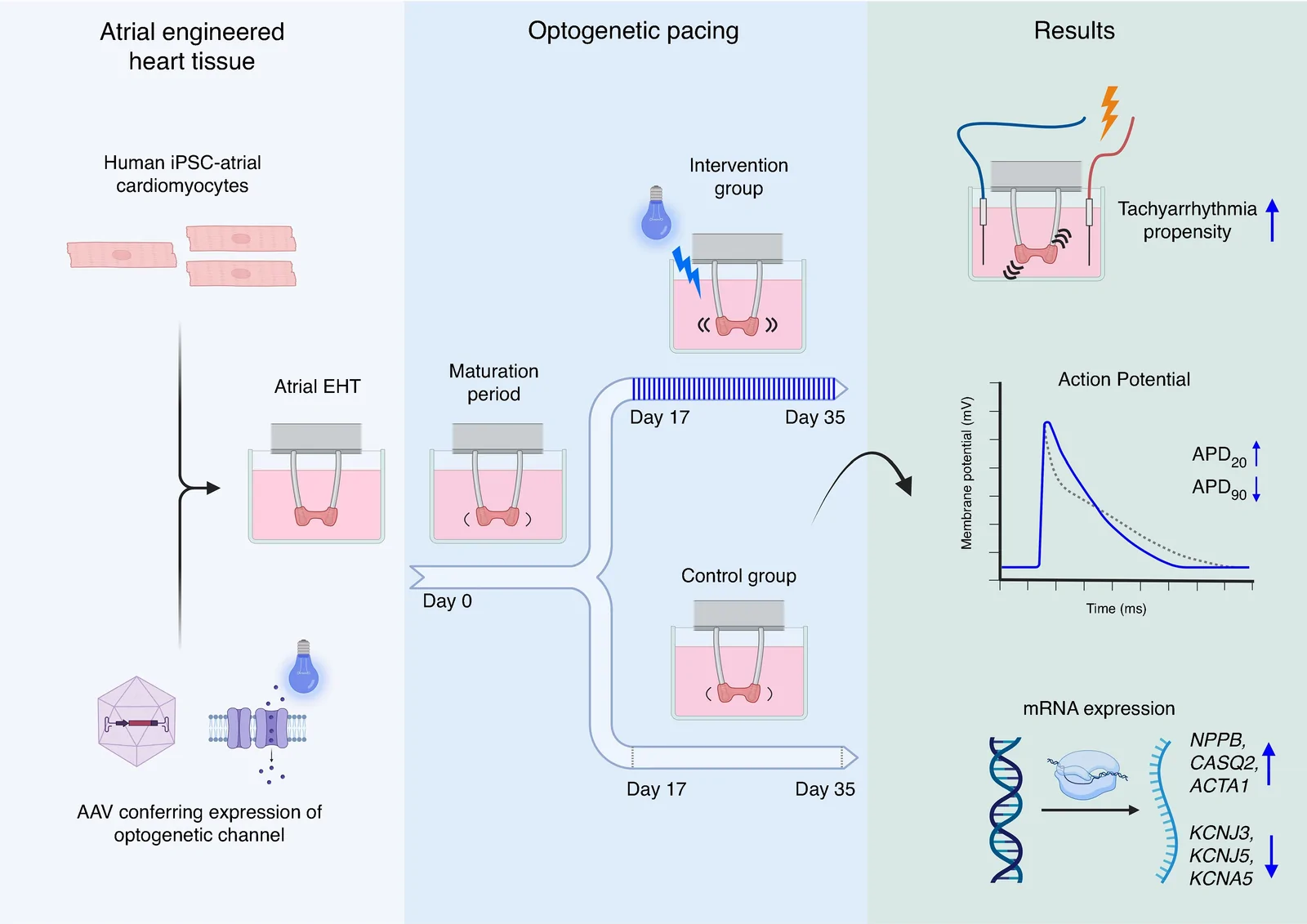

What’s new?

- Sustained fibrillation-like states can be triggered in engineered tissue constructs.
- A novel model for atrial fibrillation research without the need for an electrophysiological setup.
- Action potential conditioning from atrial-like to atrial fibrillation-like *in vitro*.

## Introduction

Chronic remodelling in atrial fibrillation (AF) has been extensively studied in atrial tissues collected from patients undergoing open heart surgery. Most mechanism-related AF research, however, relies on animal models or cell cultures.^1–3^ Animal models have the potential to faithfully reproduce almost all aspects of AF pathophysiology, including fast beating rate, cardiomyocyte stress through variable load, macro-re-entry mechanisms, and self-sustainability of the arrhythmia. Rodents are popular and rather cheap laboratory animals, but AF induction is challenging, and few laboratories have established the necessary methods.^4^ Atrial fibrillation can be more easily induced in larger animals. However, larger animal models are expensive and often ethically controversial and provide low throughput; additionally, most animals do not naturally develop AF.^3^ In addition, even large animals may differ in their atrial electrophysiology from humans, as illustrated by opposite responses to investigational atrial-selective potassium channel blockers.^5–7^ Classical monolayer cell culture models, on the other hand, are cost-effective and ethically sound and allow for medium- to high-throughput research. However, they lack anatomical and physiological features of mammalian hearts and are limited in AF modelling capabilities.^2^

As an intermediate between these two poles, atrial engineered heart tissue (aEHT) based on human induced pluripotent stem cell-derived atrial cardiomyocytes (hiPSC-aCM) differentiated in the presence of retinoic acid recapitulates key physiological properties of the human atrium.^8,9^ Engineered heart tissue can be combined with chronic or acute optogenetic pacing. A prolonged culture period over several weeks should allow the study of adaptation to rapid pacing on a mid-term scale. Successful recapitulation of AF-related changes on a molecular and cellular level could increase the arrhythmia propensity of the tissue constructs (self-sustainability of the arrhythmia, or ‘AF begets AF’^10^). Among the induced changes are alterations in aEHT electrophysiology, which can be studied by using conventional sharp microelectrode action potential (AP) recordings.

Transfer of EHT or any tissue or construct to an organ bath usually represents a final experimental step since sterility is lost. In addition, AP recordings require specialized skills, time, and effort.^11^ Moreover, in previous attempts to model fibrillation in 3D models *in vitro*, key aspects of AF-related changes and importantly induction of sustained arrhythmia were often missing. Thus, we set out to create a simplified *in vitro* AF model, by combining our experiences with hiPSC-based human atrial tissue engineering^12,13^ and optogenetics in tissue constructs.^14,15^ Optogenetics was used for chronic tachypacing, to pre-condition aEHTs and facilitate later induction of episodes of sustained, fast, incoherent beating by electrical burst stimulation. In contrast to earlier approaches, assessment of tachyarrhythmia inducibility was simplified and now based on contractility analysis rather than AP recordings. Many of the employed methods are scalable and adaptable to different research settings and questions. We thus believe that our experience could render tachyarrhythmia as a readout more accessible for AF research.

## Materials and methods

### Human induced pluripotent stem cell culture and cardiac differentiation

For all hiPSC work, a published cell line obtained from a healthy donor, UKEi001-C was employed (registered at hpscreg.org). The cell line was established by the UKE Stem Cell Core Facility, using the Sendai virus-based CytoTune kit (Life Technologies). Human iPSC culture was based on DMEM/F12 medium supplemented with bFGF, TGF-β1, dorsomorphin, and activin A (FTDA medium). Atrial-like cardiac differentiation of hiPSC was performed as described previously, combining protocols published by Devalla *et al.*, Krause *et al.*, and Breckwoldt *et al.*^12,16,17^ The differentiation protocol has three phases: embryoid body (EB) formation, mesoderm induction, and cardiac differentiation. Embryoid body formation was achieved by hiPSC culture in spinner flasks with constant stirring for 24 h. Mesoderm formation was induced by exposure to BMP-4 (3 ng/mL), activin A (2 ng/mL), and bFGF (5 ng/mL). Cardiac differentiation was induced after 3 days by inhibition of Wingless Int-1 (WNT) signalling (XAV-939, 1 µM). During this step, atrial fate was achieved by supplementation with 1 µM retinoic acid, supplied unfiltered in dimethyl sulfoxide (DMSO).^8^ Embryoid bodies were finally dissociated with collagenase II (200 U/mL) to obtain single cardiomyocytes for aEHT generation. Expression of the cardiac marker cardiac troponin T (cTNT) was assessed by flow cytometry for quality control, and only differentiation batches with more than 90% of cTNT^+^ cells were used. If necessary, magnetic-activated cell sorting (Miltenyi) was applied to increase the percentage of cTNT^+^ cells accordingly.

### Engineered heart tissue generation and culture

**Figure 1:**
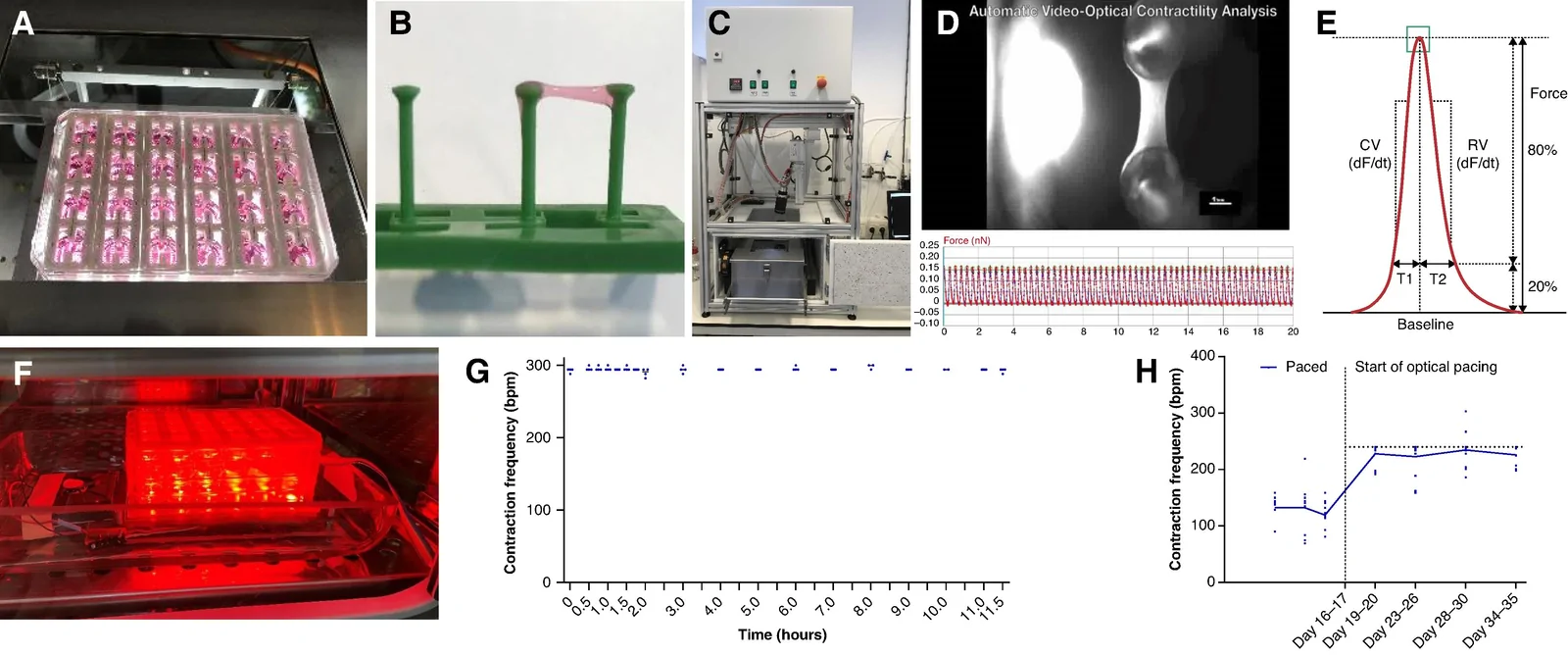
Engineered heart tissue culture, video-optical analysis, and optogenetic pacing. (*A*) Engineered heart tissues in 24-well culture format. (*B*) Single EHT attached to silicone posts. (*C*) Glass-top incubator with video-optical recording system. (*D*) Video-optical recording of single EHT and respective force trace, analysed via pattern recognition software. (*E*) Force peak and selection of analysed parameters. (*F*) A 24-well plate with silicone EHT racks on top of tri-colour LED array. The arrangement of LEDs matches the 24-well plates used for aEHT culture. Red light was used for illumination during video-optical recording, whereas blue light was used for optical pacing. The radial fan on the left was used to expel excess heat. (*G*) CheRiff2.0 optogenetic pacing adherence. For 12 h, aEHTs (*n* = 4) transduced with CheRiff2.0 were subjected to continuous optical pacing at a frequency of 5.0 Hz and pulse duration of 40 ms. Vertical axis displays the actual contraction frequency of aEHTs as repeatedly measured via video-optical recording. (*H*) Pacing adherence of CheRiff2.0-aEHTs over time. Each dot represents a single EHT. Horizontal dotted line represents a pacing frequency of 4 Hz (240 b.p.m.). Continuous line represents mean contraction frequency. N = 9–16 aEHTs from three batches. aEHT, atrial engineered heart tissue; LED, light-emitting diode.

Generation of fibrin-based human aEHTs in 24-well format from 1 × 10^6^ hiPSC-aCM each was carried out according to published protocols.^12,16^ In brief, hiPSC-aCM were either dissociated freshly after differentiation or thawed, re-counted, and used to cast aEHT. To this end, cells were resuspended in DMEM containing 10% horse serum, 1% L-glutamine, 1% penicillin/streptomycin solution, and 5 mg/mL fibrinogen. For optogenetic pacing, adeno-associated virus (AAV) transduction was carried out by adding the virus vector to the casting mixture. After addition of 3% thrombin for polymerization, the mixture was quickly transferred into pre-formed agarose casting moulds in 24-well cell culture format, fitted with silicone mounting racks. After ∼2 h of polymerization, the mounting racks with the attached aEHTs were transferred to a fresh 24-well cell culture plate containing standard aEHT medium (DMEM supplemented with 1% penicillin/streptomycin, 10% horse serum, 10 µg/mL insulin, and 33 µg/mL aprotinin), for long-term culture (*Figure* *1A* and *B*). After an initial maturation period of 17–18 days with three medium changes per week, during which aEHTs acquired spontaneous, regular coherent beating activity and force reached a plateau value, optogenetic pacing was initiated. Atrial EHT contractility was regularly analysed by video-optical recording, using analysis of silicone mounting post-deflection to calculate force in an automated manner (*Figure 1C–E*; hardware: EHT Technologies, DiNAQOR; software: Consulting Team Machine Vision). After each medium change, aEHTs were allowed 1 h to readjust before contractility measurement. After this period, spontaneous beating of both chronically paced and unpaced aEHTs was measured without pacing. Subsequently, pacing was re-initiated in the pacing group, and paced aEHT contractility was measured once more. Contractility of aEHTs from different batches was not measured on the exact same time points over the course of their development, but within a range of 1–3 days. Only aEHTs developing a baseline force of at least 0.01 mN were included in the subsequent analyses.

During episodes of the fibrillation-like state, automated peak detection by the software was impaired, so a different approach was taken to analyse contraction frequency and relative resting length during the episodes: For analysis of contraction frequency during arrhythmia, single contraction peaks were visually identified and counted over a period of at least 10 s; frequency was then calculated. For analysis of relative resting length during arrhythmia, silicone post-deflection recognition threshold of the software was gradually decreased in 5%-steps of maximal deflection (default setting 95%). The first percentage at which a peak was recognized by the software (e.g. 85%), was used as a surrogate for relative resting length.

### Transduction of atrial engineered heart tissue and choice of optogenetic channels

For optical pacing, different light-excitable ion channels were evaluated. Using CRISPR/Cas9 genome editing, we first created a cell line stably expressing *Volvox carteri* channelrhodopsin 1 (VChR1^18^) under the control of a CAG promoter. Genome editing in the cell line UKEi001-C was carried out as previously described using published guide RNAs.^19,20^ For further experiments, aEHT was transduced upon casting with different AAVs encoding optogenetic cation channels. Generation of recombinant AAV6 particles was carried out as previously described.^21^ In brief, AAV transfer plasmid, pAAV-RC6 pHelper, was transfected into HEK293T cells (Cell Biolabs). After harvesting, washing (PBS), three freeze–thaw cycles, and Benzonase treatment, cell debris was pelleted, and vector-containing lysates were purified using Iodixanol step gradients. The genomic titres of DNase-resistant recombinant AAV particles were determined by quantitative PCR using woodchuck hepatitis virus post-transcriptional regulatory element (WPRE)-specific primers (WPRE-F 5´-ccgttgtcaggcaacgtg, WPRE-R 5´-agctgacaggtggtggcaat). Adeno-associated virus transfer plasmid was employed as a copy number standard. Adeno-associated viruses were then added to the aEHT casting mixture as described above. Three AAV6 constructs were generated, containing the coding sequence for either Chronos,^22^ CheRiff2.0 (see Supplementary material online, *Figure S1*), or PsCatCh2.0f^23^ under the control of a cTNT promoter, coupled to the enhanced yellow fluorescent protein (eYFP) to allow for fluorescence-based detection and analysis of transduction efficiency. CheRiff2.0 was engineered by adding an artificial signal peptide Lucy-Rho to CheRiff.^24^ CheRiff2.0 showed enhanced expression and photocurrent after expression in *Xenopus laevis* oocytes (see Supplementary material online, *Figure S1*) following the method described in Duan *et al.*^25^ Through several consecutive batches of transduced aEHT and subsequent flow cytometry analysis after papain-based aEHT dissolution and live/dead staining (10 U/mL papain, 1 mM EDTA, and 5.5 mM L-cysteine-HCl in 1 × EBSS, 20–40 min at 37°C; LIVE/DEAD Fixable Far-Red Dead Cell Stain kit, Thermo Fisher), MOI was titrated to ∼70–90% of YFP + cells (see Supplementary material online, *Figure S2*). Subsequent experiments were carried out at the resulting calculated MOI of 300 for Chronos, 750 for CheRiff2.0, and 700 for PsCatCh2.0f, based on virus titre determination as described above.

### Components of the pacing device

To allow for optogenetic pacing of aEHT, we constructed custom-designed optogenetic hardware. The setup was based on a printed circuit board (JLCPCB), mounted with 24 addressable tri-colour light-emitting diodes (RGB-LEDs, 5 mm SMD). This light-board (*Figure 1F*) was positioned below the 24-well cell culture plate containing aEHTs, both for long-term pacing and video-optical analysis. Light-emitting diodes were controlled by a programmable microcontroller (Arduino A000005, Nano ATMega328) and custom-programmed software (Arduino IDE, code available at https://github.com/owlcap0ne/pacer_led_board). As all employed opsins are well excitable using blue light of 465 nm wavelength (0.19 mW/mm^2^), blue LEDs were used for channel excitation. As all employed opsins should remain silent at red light, red LEDs (625 nm, 0.1 mW/mm^2^) were used for background illumination during video-optical recording. Light intensity was quantified using a Thorlabs PM400 power meter device in the dark. Measurement was carried out three times per condition, integrating the light signal over 1 cm^2^ in contact with the surface of a 1.7 mm thick transparent polystyrene plate, which was used as spacer between the 24-well plate carrying the EHTs and the LED-bearing circuit board. This plate was used to thermally isolate the EHT plate from the LED board (the space between the two plates was actively ventilated with a 5 V miniature radial ventilator for EHT culture). Light intensity was quantified at 465, 525, and 625 nm illumination, directly above the LED, i.e. in a similar position as the respective illuminated EHT, in ∼2.5 mm total distance from the LED (see values above; green light: 0.23 nW/mm^2^).

### Electrical burst pacing

For electrical burst pacing, aEHT racks were fitted with carbon pacing electrodes (EHT Technologies, DiNAQOR) as previously described.^26^ To induce episodes of arrhythmia, 0.5 s trains (‘bursts’) of fast biphasic pulses of 4 ms duration per pulse and 2–4 V at 20 Hz were individually triggered and applied to the tissues under continuous video-optical recording. If a single electrical burst failed to trigger arrhythmia, burst application was repeated up to 20 times. If no arrhythmia occurred after the 20th burst, the respective aEHT was categorized as displaying no arrhythmia at all.

### Optical mapping analysis

Optical mapping was carried out using a custom-built setup comprising a gas and temperature-controlled bath and a Teledyne Kinetix high-speed back-illuminated sCMOS camera (Cairn research). Micromanager software (ImageJ^27^) and ElectroMap^28^ software were used for image acquisition and analysis, respectively.

For optical mapping of EHTs, dye loading was performed by individually incubating aEHTs in 2 mL pre-warmed FluoroBrite DMEM containing the red-shifted voltage sensitive dye RH237 (Santa Cruz Biotechnology, 10 µM) for 10 min (transduced aEHT) or 45 min (untransduced aEHT), respectively at 37°C and 5% CO_2_ in a 24-well plate. The system was filled with 37°C pre-warmed FluoroBrite DMEM (Gibco), and EHTs were placed at the bottom of the bath, attached to their silicone mounting posts in an inverted manner. Imaging was performed under 5 mL/min continuous flow rate of the media. The initial rhythm state of the EHTs was monitored optically in live camera view. For mapping of aEHTs expressing CheRiff2.0 and eYFP, a black background was used, to reduce background light reflection. For dye excitation, a combination of a CoolLED pE-4000 light source, a fibre-optic wire, and an attached light diffusor—made from a cube of acrylic glass which was placed directly in front of the light source—was used. The light source was set to 635 nm wavelength and 7% intensity. Camera acquisition was carried out using a suitable filter. For mapping of untransduced aEHTs, the Cairn MacroLED Illumination System with two separate LED heads was used for dye excitation.

In CheRiff2.0 aEHTs, to reduce movement, a total of 100 µL 34 mM -(-)blebbistatin (TargetMol) was pipetted directly into the superfusion chamber. In the uncoupled state, 8-bit images with frame rates of ∼995 frames per second and 10 000 counts at 1 ms exposure time and 4 × 4 binning were acquired (the sensor pixel size was 6.5 µm according to the manufacturer). Pixel size was calculated for these binning factors and adjusted in the ElectroMap software along with the frame rate of the camera for each optical mapping acquisition. Pacing, burst pacing, and induction of burst-evoked episodes of fibrillation were performed as described.

For untransduced aEHTs, pacing threshold was determined for each EHT individually. Starting from 0.4 V, voltage was increased until the aEHT followed pacing. Double pacing threshold was used for analyses (1.2–3 V). Asymmetric electrical pacing units were placed at the left post of the aEHT,^29^ and pacing was carried out at 3, 4 and 6 Hz. During continuous pacing with 3 Hz, the aEHTs were again electromechanically uncoupled by adding 100 µL 34 mM -(-)blebbistatin into the superfusion chamber, resulting in a total concentration of 11 µM in the chamber. After 5 min of incubation with blebbistatin, aEHT contraction was terminated. In the uncoupled state, 16-bit images with frame rates of ∼400 frames per second and 10 000 counts at 2 ms exposure time and 4 × 4 binning were acquired. During acquisition, pacing capture was checked by using a live region of interest (ROI) plotter embedded in MicroManager^27^ supplied by customScopes. For further analysis, the TIFF-format files were binned further by a factor of 4 and converted into MATLAB files. In ElectroMap, at least 50 sequential beats were averaged in order to improve signal-to-noise ratio. Analysis was performed using a Gaussian spatial filter [5 × 5 pixel area, standard deviation (σ) = 1.5] with Savitzky–Golay filtering. Activation times were measured as the time of depolarization midpoint. Conduction velocity (CV) was calculated from the isochronal maps using the polynomial multi vector method with a 2 × 2 pixel area, due to the small width of an aEHT. Start of AP duration was measured from AP Peak.

All analyses were carried out on a defined ROI, excluding the post region to avoid regional autofluorescence.

### Action potential recording

Action potential recordings were carried out using the sharp microelectrode method as described previously.^11^ To this end, after 35 days in culture, aEHTs were pinned down in a superfused bath containing 127 mM NaCl, 5.4 mM KCl, 1.5 mM MgCl_2_, 1.8 mM CaCl_2_, 10 mM glucose, 22 mM NaHCO_3_, and 0.42 mM NaHPO_4_, under calibration with O_2_/CO_2_ (95:5), 36.5 ± 0.5°C, pH 7.4. Action potentials were analysed off-line with Lab-Chart software (ADInstruments, Dunedin, New Zealand).

### Atrial engineered heart tissue immunofluorescence

For immunofluorescence, aEHTs were fixed in 4% paraformaldehyde (PFA) solution for 12–24 h and subsequently embedded in 4% agarose. The samples were then sectioned into 70 µm slices using a vibratome. The sections were incubated in blocking buffer [1% bovine serum albumin (BSA), 0.3% Triton X-100] for 60–90 min at room temperature. Afterwards, the primary antibody (anti-α-actinin, mouse, 1:500) was added and incubated at 4°C for 12–24 h. The solution from the sections was then aspirated, and sections were washed three times with PBS and subsequently incubated with the labelled secondary antibody (AF633 anti-mouse, 1:800) and 4′,6-diamidino-2-phenylindole (DAPI) for nuclear staining (1:1000) for 60–90 min at 4°C. Finally, the sections were washed three times with PBS and mounted on glass slides for imaging. CheRiff2.0 was visualized using its eYFP-tag.

### Gene expression analysis

Gene expression analysis was performed by standard real-time (RT)-qPCR and bulk RNA sequencing. For RT-qPCR, aEHTs were halved after culture, snap frozen, and stored at −80°C. RNA was extracted from frozen tissue, and 200–500 ng was reverse transcribed (Applied Biosystems); the resulting cDNA was subjected to SYBR Green-based qPCR (Solis BioDyne) after 6- to 10-fold dilution. Primers can be found in Supplementary material online, *Table T1*. Exploratory gene expression analysis by quantitative RNA sequencing was carried out according to established procedures by the Genomics Core Facility of Münster University (Münster, Germany). RNA was quality controlled (Agilent Bioanalyzer), and only RNA with an RNA integrity number (RIN) value of >6 was used. Library preparation was carried out with the Illumina TruSeq Stranded mRNA kit, and libraries were sequenced in paired-end mode (HiSeq, Illumina), read length 151 bp, and ∼25 million reads per sample. Data analysis was carried out by the UKE Bioinformatics Core Facility. For this, sequence reads were processed with fastp (v0.23.2) to remove sequences originating from sequencing adapters and sequences of low quality using the programme’s default parameters.^30^ Reads were then aligned to the human reference assembly (GRCh38.110) using STAR (v2.7.10a).^31^ Differential expression was assessed using DESeq2 (v1.36.0).^32^ A gene was considered significantly differentially expressed if the corresponding absolute value of the log_2_-transformed fold change (log_2_FC) was 1 or larger and, in addition, the false discovery rate (FDR) did not exceed a value of 0.1.

### Protein analysis by Western blot

Western blot was performed according to established procedures. Antibodies were directed against Ca^2+^/calmodulin-dependent protein kinase II (CaMKII; 1:1000; BD Transduction Laboratories #611292), CaMKII phosphorylated at Thr286 (1:1000; Cell Signaling #12716), cardiac calsequestrin 2 (CASQ2; 1:1000; Proteintech #18422-1-AP), and protein kinase B (PKB; Akt, 1:400; Cell Signaling #9271) phosphorylated at Ser473. Protein was extracted from snap-frozen halved aEHTs using 50 µL of M-PER extraction buffer (Thermo Fisher) per tissue, supplemented with PhosSTOP phosphatase inhibitor (Roche). Tissue lysates were separated on 15% acrylamide containing SDS-PAGE gels and blotted onto nitrocellulose membranes. Blocking was carried out in 10% milk for 1 h at 4°C, before incubation with the primary antibody at 4°C overnight and subsequent incubation with horseradish peroxidase (HRP)-coupled secondary antibody for 1 h. Horseradish peroxidase signal was visualized with Pierce ECL substrate (Thermo Fisher).

### Statistics

Statistical analyses were performed as indicated in the respective figure with the Prism software (GraphPad Software, Boston, USA). If not indicated otherwise, error bars represent standard deviation.

## Results

### Manufacturing of the pacing device and optogenetic excitability

For optogenetic pacing in 24-well culture fibrin-based aEHT, the expression level of a suitable optically excitable channel as well as respective hardware needed to be established. We manufactured a custom-designed optogenetic pacing device from readily available low-cost components. The hardware consisted of a printed circuit board, the dimensions of which allowed it to exactly fit below a standard 24-well cell culture plate. Each well was equipped with an addressable microarray of red, green, and blue micro-LEDs (*Figure 1F*). Light-emitting diodes were addressed to flash or glow as desired with an attached programmable Arduino-type microcontroller. Constant red light (620 nm) was used for illumination during video-optical contractility analysis, whereas pulsed blue light (465 nm) was used for optogenetic excitation.

The hardware was used to evaluate the suitability of different optogenetically non-selective cationic channelrhodopsins at different expression levels for optogenetic pacing.

### Application of optogenetic pacing to atrial engineered heart tissue

First attempts to optogenetically pace aEHT were carried out with hiPSC-aCM stably expressing VChR1. We evaluated whether red light-mediated activation of the channel would interfere with blue-light pacing and depolarize aEHT, by analysing contractility of aEHT at constant red illumination of different intensities and detected no differences depending on red illumination (see Supplementary material online, *Figure S3*). Thus, all subsequent video-optical contractility analyses of aEHT expressing VChR1, or other optogenetic channels with similar (or more blue-shifted) excitation spectra, were carried out with red illumination. The optogenetic pacing itself was performed with flashing blue LEDs. *Volvox carteri* channelrhodopsin 1-expressing aEHT proved not ideal for longer-term optogenetic pacing, as it was subject to rapid desensitization, usually within minutes, which necessitated regular pacing breaks^33^ (see Supplementary material online, *Figure S4*). We therefore set out to compare additional optimized channelrhodopsins and used AAV6 as a vector to express Chronos, CheRiff2.0, or PsCatCh2.0f, respectively. We had previously optimized CheRiff2.0 for better membrane integration, resulting in 5.5-fold higher photocurrents, compared to CheRiff (see Supplementary material online, *Figure S1*). Through transduction of aEHT with different amounts of AAV, we titrated expression of the channels to similar levels as assessed by eYFP fluorescence (70–90% of cells), to allow for direct comparisons. CheRiff2.0 was the only channel that allowed for longer-term pacing at nearly complete pacing capture fidelity (*Figure* *1G* and *H*; Supplementary material online, *Figures S5*–*S6*). Neither of the channelrhodopsins, PsCatCh2.0f and Chronos, was useful to pace aEHTs at frequencies significantly higher than their respective baseline contraction frequency (see Supplementary material online, *Figure S5*). Furthermore, force development in CheRiff2.0-transduced aEHTs was similar to untransduced control aEHTs in most batches (see Supplementary material online, *Figure S6*).

**Figure 2:**
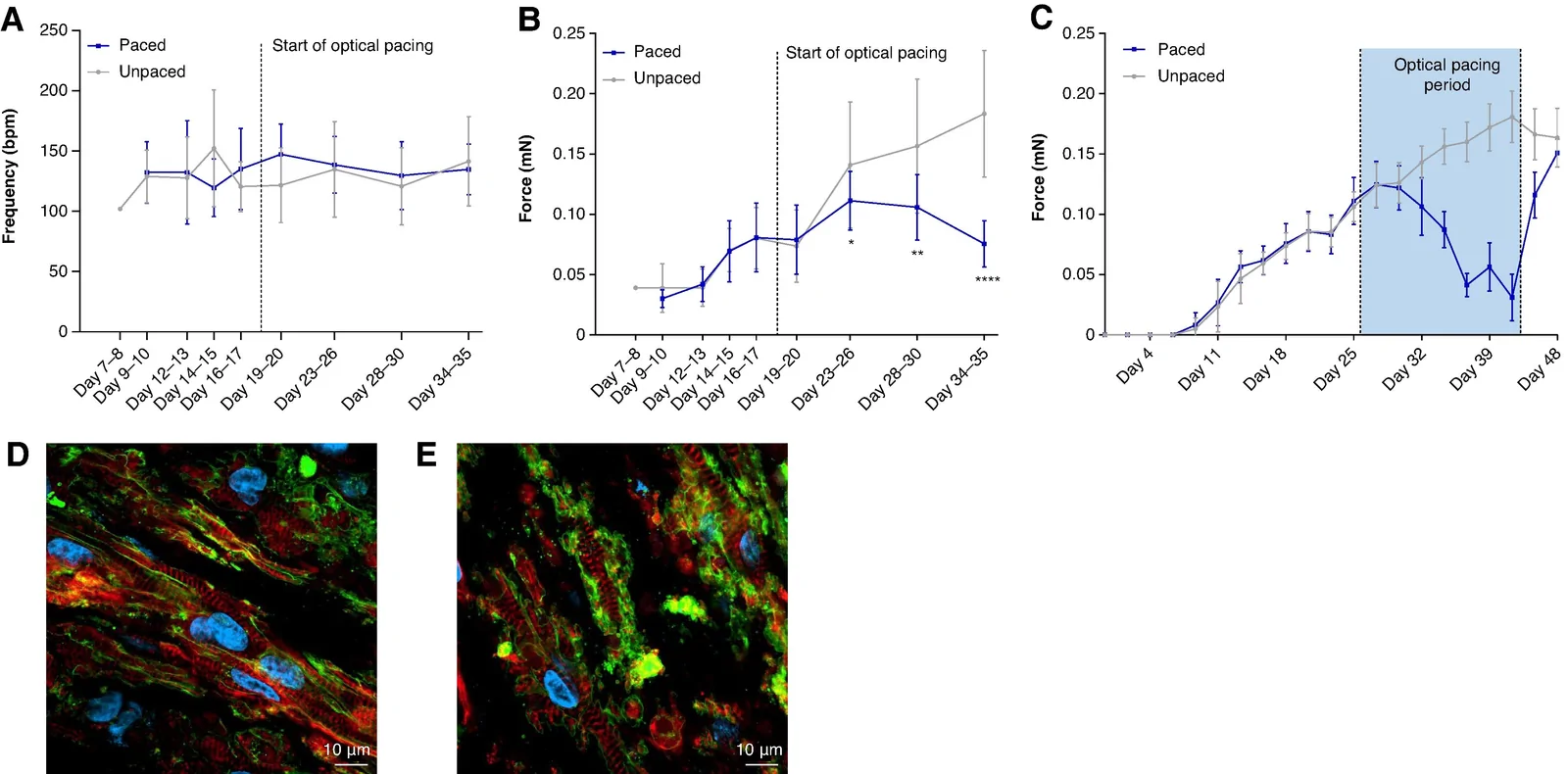
Development of aEHT spontaneous contractility parameters over time, immunofluorescence imaging. (*A*) CheRiff2.0-expressing aEHTs. Spontaneous contraction frequency (i.e. recorded without pacing) showed no significant differences between paced and unpaced groups. (*B*) CheRiff2.0-expressing aEHTs. After the start of optical pacing, spontaneous contraction force of chronically paced aEHTs increased more slowly and ultimately decreased significantly compared to unpaced controls. *A* and *B*; N = 13–14 aEHTs per group from three batches, multiple linear regression, *P < 0.05, **P < 0.01, ****P < 0.0001. (*C*) VChR1-expressing aEHTs. From Day 28 to 45, aEHTs were subjected to intermittent optical fast pacing. After termination of optical pacing, contraction force returned to the level of unpaced control aEHTs. N = 32–33 aEHTs from two batches. (*D* and *E*) Confocal microscopy immunofluorescence imaging of a non-paced control aEHT (*D*) and a paced aEHT (*E*). DAPI (blue), eYFP (green), and alpha-actinin (red). Representative image, scale bar 10 µm. eYFP fused to CheRiff2.0, revealing robust membrane-bound expression of CheRiff2.0. aEHT, atrial engineered heart tissue; eYFP, enhanced yellow fluorescent protein; VChR1, *Volvox carteri* channelrhodopsin 1.

Tachypacing led to an impairment of force development, but no change of spontaneous contraction frequency. Force impairment was fully reversible after pacing termination (*Figure 2A–C*; Supplementary material online, *Figure S5B*). Confocal immunofluorescence imaging at the end of culture revealed robust, membranous expression of CheRiff2.0. Sarcomere structure was well organized in unpaced control aEHTs, while paced aEHTs displayed sarcomeric disarray (*Figure* *2D* and *E*; Supplementary material online, *Figure S7*). However, reversibility of force decline argued against cell death (*Figure 2C*; Supplementary material online, *Figure S5B*).

### Long-term optogenetic pacing increases propensity for tachyarrhythmias in atrial engineered heart tissue

Burst-induced sustained arrhythmia has been described in ventricular EHT (vEHT) conditioned with intermittent tachypacing.^14^ We thus aimed to similarly condition aEHT and evoke fibrillation-like episodes by electric burst pacing.

Atrial EHTs expressing CheRiff2.0 were either continuously tachypaced at 4 Hz and 40 ms optical pulse duration for 17–18 days or left unpaced, exceeding previous endeavours for longer-term tachypacing.^34^ At the end of the optogenetic pacing period, electrical burst pacing was applied to the aEHTs to induce arrhythmias. Using carbon electrodes, fast trains (‘bursts’) of biphasic electrical pulses of 4 ms duration per pulse at 2–4 V and 20 Hz with a burst duration of 0.5 s were repeatedly applied to the aEHT up to 20 times under continuous video-optical recording. If burst application was successful, recording was continued for up to several hours (see below).

**Figure 3:**
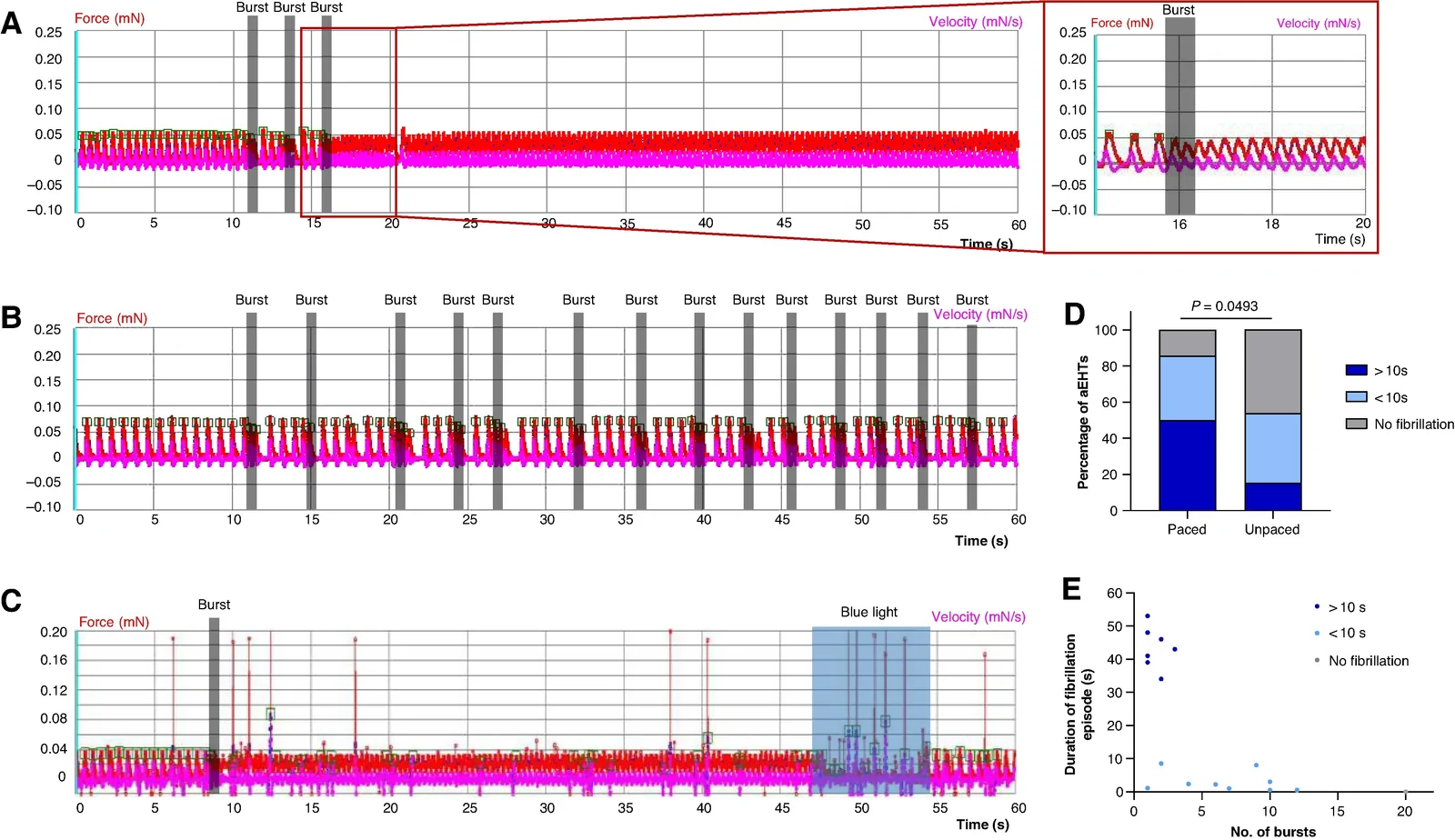
Electrical burst pacing of aEHTs. (*A–C*) Exemplary original recording traces of aEHTs during electrical burst pacing. (*A*) Optogenetically paced aEHT trace. Burst pacing was applied three times. Note fibrillation-like pattern after the third burst, with higher beating frequency and decreased contractile force. Impaired relaxation led to insufficient beat capture by the recording system, as indicated by the lack of peak recognition squares in this part of the trace. Inlay: Higher magnification of trace A in the moment the fibrillation-like episode was triggered. (*B*) Unpaced control group aEHT, no fibrillation-like episode despite repeated electrical burst application. (*C*) Fibrillation was triggered in this paced aEHT with the first electrical burst. After 48 s, a 6 s pulse of constant blue-light illumination was applied. This pulse caused the aEHT to convert back to its regular spontaneous beating pattern. (*D*) Duration of fibrillation-like episodes of optogenetically paced aEHTs and unpaced controls. Longer fibrillation-like episodes (>10 s) were significantly more prevalent in the pacing group. Fibrillation-like episodes could last up to several minutes before converting back into regular beating pattern. N = 13–14 aEHTs per group from three batches, multiple logistic regression. (*E*) Duration of fibrillation-like episode in relation to the number of bursts needed to trigger the fibrillation-like state. This analysis revealed the existence of three distinct phenotypes: Atrial EHTs that showed persisting arrhythmia, self-limited arrhythmia under 10 s, or no arrhythmia. In aEHTs with persisting arrhythmia, no more than three bursts of electrical field stimulation were necessary to evoke this fibrillation-like state. Correlation coefficient = −0.77 (Kendall’s tau *b*, *P* < 0.00001). N = 13–14 aEHTs per group from three batches. aEHT, atrial engineered heart tissue.

Electrical bursts were indeed often followed by beating pattern that was characterized by fast, acutely weaker, and incoherent contractions with impaired relaxation and a ‘wobbly’ phenotype (*Figure 3A*; Supplementary material online, *Video V1*). In the following, we call this phenomenon ‘fibrillation-like’, although the recorded contractions did not always appear strictly arrhythmic. This is the first time that this phenomenon could be shown in aEHT. During these ‘burst-evoked episodes of fibrillation’ (BEEF), the mean contraction frequency was 192 b.p.m., compared to a baseline frequency of 140 b.p.m. in a regular beating pattern immediately before burst stimulation (*P* = 0.0007, *n* = 12 aEHTs from five batches, paired *t*-test; Supplementary material online, *Figure S8A*). The relative resting length of the aEHTs during BEEF on average was only 94.6% of that of aEHTs with a regular beating pattern (*P* = 0.0322, *n* = 13 aEHTs from five batches, ratio paired *t*-test; Supplementary material online, *Figure S8B*).

Interestingly, we observed a significantly higher propensity for persistent episodes of the fibrillation-like state (>10 s) in aEHTs which previously had undergone tachypacing, compared to unpaced aEHTs (50.0% vs. 15.4%, *P* = 0.0493, *n* = 13–14 aEHTs per group from three batches, multiple logistic regression; *Figure 3A–D*). Moreover, analysis of the duration of the episodes of arrhythmia in relation to the number of bursts required to induce arrhythmia revealed a negative correlation of the two parameters with a Kendall’s tau correlation coefficient of −0.77 (*P* < 0.00001; *Figure 3E*): The fewer bursts were required to trigger BEEF, the longer the duration of the episode lasted, emphasizing the arrhythmia propensity of the respective aEHT.

Evoked episodes of arrhythmia showed a wide range of duration, but could in general be classified as one of two phenotypes: Type 1 BEEF would last for <10 s, as shown in *Figure* *3D* and *E* (*n* = 13 from four batches). Type 2 BEEF was a more ‘stable’ arrhythmia; its maximal duration was systematically assessed in *n* = 4 aEHTs. In these aEHTs, BEEF duration was at least 1 h; the longest BEEF duration we observed was 4 h and 45 min. Type 2 BEEF did not terminate spontaneously; measurements were ended only when contractile force was too low for the software to track, or when BEEF was terminated by applying blue light (see Supplementary material online, *Figure S9*). An ∼100 ms period of illumination was sufficient to reliably terminate BEEF (*Figure 3C*). In some aEHTs, a blue light impulse as short as 10 ms was sufficient to terminate BEEF, suggesting the existence of a vulnerable phase within the arrhythmia cycle. Prolonged illumination (6 s were repeatedly evaluated) led to constant activation of CheRiff2.0 with a subsequent depolarization block and no contractility (*Figure 3C*, only pattern recognition software noise during constant illumination).

### Characterization of the arrhythmic state

**Figure 4:**
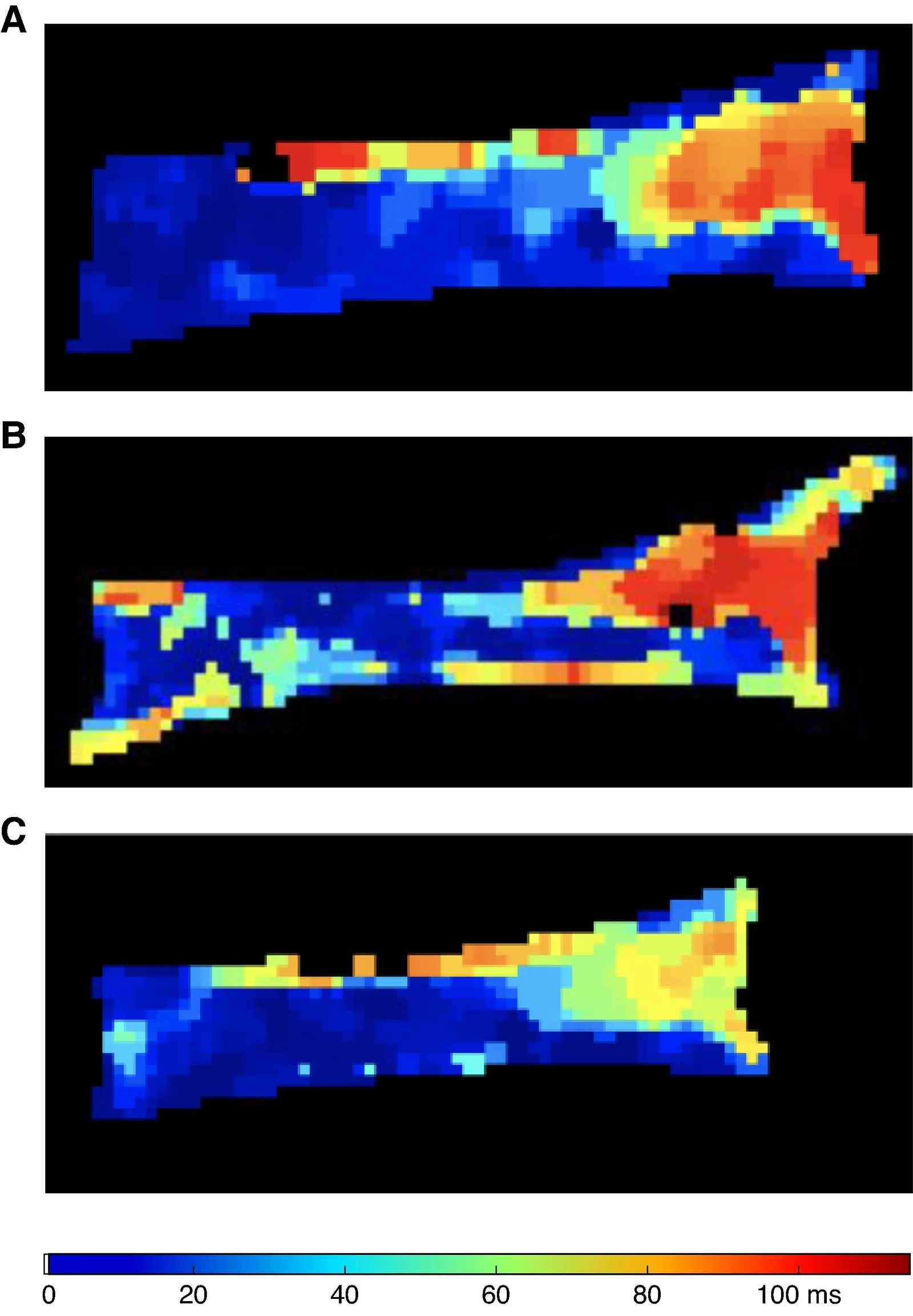
Optical voltage mapping with RH237 at 633 nm illumination. Maps showing the propagation of activation. Colour codes for the time at which depolarization midpoint was reached for each pixel, averaged over 10 s (20–35 contraction cycles, depending on frequency), (*A*) for intrinsic activation, (*B*) for burst-evoked episodes of fibrillation (BEEF), and (*C*) for activation during 3 Hz electrical pacing after termination of BEEF, respectively.

To characterize the physiological nature of the BEEF, we performed optical mapping analysis with the red-shifted voltage dye RH237 (see Supplementary material online, *Figure S10*). During spontaneous beating and regular electrical pacing, each aEHT displayed one specific centre of automaticity from which activation travelled through the tissue (*Figure* *4A* and *C*; Supplementary material online, Figure  *S10* and *Video V2A* and *C*). In the optical mapping system, BEEF could be robustly evoked in the same manner as in our bench-top incubator-equipped analysis system. After BEEF induction, this regular automaticity changed to a state with uncoordinated action of several areas with pacemaker activity (*Figure 4B*; Supplementary material online, *Video V2B*). Termination with blue light reverted the excitation pattern back to its initial state (*Figure 4C*).

### Long-term optogenetic pacing induces fibrillation-like properties in atrial engineered heart tissue

**Figure 5:**
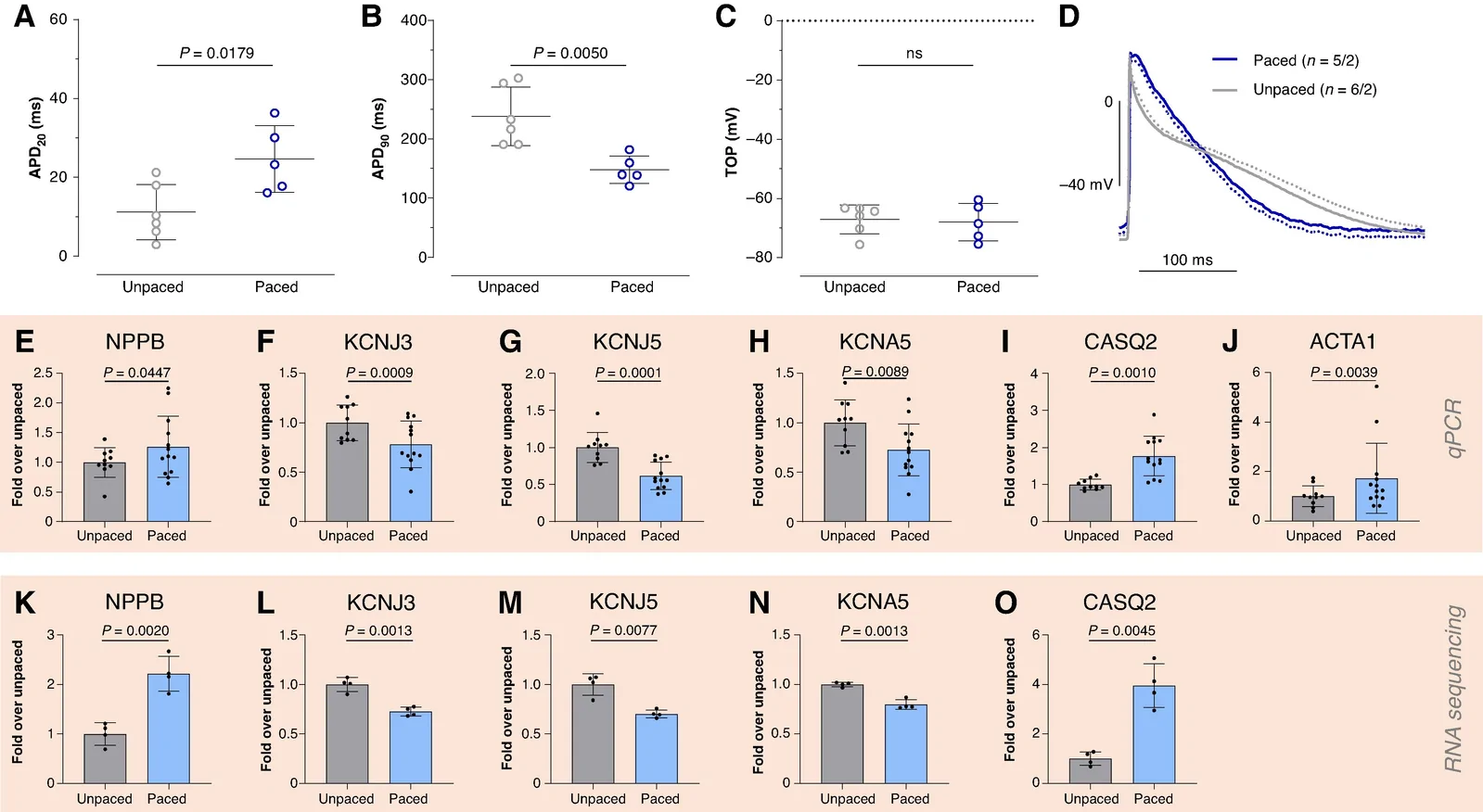
Action potential recordings and gene expression. (*A*) AP duration at 20% of repolarization (APD_20_) in paced vs. unpaced aEHTs. (*B*) AP duration at 90% of total repolarization (APD_90_) in paced vs. unpaced aEHTs. (*C*) Take-off potential (TOP) in paced vs. unpaced aEHTs. *A–C*; N = 5–6 aEHTs per group from two batches. Unpaired *t*-test. (*D*) ‘Triangularization’ of AP shape in long-term fast-paced aEHTs. Dotted line indicates 95% confidence interval of AP shape. (*E–J*) Messenger RNA abundance (qPCR) of selected genes in paced aEHTs and unpaced controls. (*E*) NPPB (brain natriuretic peptide). (*F*) KCNJ3 (G protein-activated inward rectifier potassium channel 1). (*G*) KCNJ5 (G protein-activated inward rectifier potassium channel 4). (*H*) KCNA5 (potassium voltage-gated channel, shaker-related subfamily, member 5). (*I*) CASQ2 (calsequestrin 2). (*J*) ACTA1 (actin alpha 1, skeletal muscle). *E–J*; N = 10–13 aEHTs per group from two batches, two-way analysis of variance (ANOVA) test. Correction for multiple comparisons: Holm–Šidák method, alpha = 0.05. Significance threshold after correction: *P* = 0.0013. (*K–O*) RNA sequencing data of selected genes from VChR1-transduced aEHT compared between paced aEHTs and unpaced controls. (*K*) NPPB (brain natriuretic peptide). (*L*) KCNJ3 (G protein-activated inward rectifier potassium channel 1). (*M*) KCNJ5 (G protein-activated inward rectifier potassium channel 4). (*N*) KCNA5 (potassium voltage-gated channel, shaker-related subfamily, member 5). (*O*) CASQ2 (calsequestrin 2). *K–O*; N = 4 aEHTs per group from one batch, *t*-test with Welch’s correction. Correction for multiple comparisons: Holm–Šidák method, alpha = 0.05. Significance threshold after correction: *P* = 0.009. In complete RNA-sequencing dataset, all differences significant after FDR adjustment. aEHT, atrial engineered heart tissue; AP, action potential; FDR, false discovery rate; VChR1, *Volvox carteri* channelrhodopsin 1.

After successful recapitulation of the ‘AF begets AF’ phenomenon, we aimed to elucidate the underlying mechanisms of pre-conditioning. As mentioned above, aEHTs were continuously optogenetically tachypaced for 17–18 days. Contractility was analysed in regular intervals both without pacing for all aEHTs (data not shown) and with pacing in the respective paced group, to monitor full pacing capture (*Figure* *1G* and *H*). At the end of the culture period, sharp microelectrode AP recording was carried out. In tachypaced aEHT, AP duration at 20% of repolarization (APD_20_) was prolonged (*Figure 5A*), but AP duration at 90% of repolarization (APD_90_) was shortened (*Figure 5B*), resulting in a triangularization of the AP shape (*Figure 5D*), a phenomenon canonically observed in human atrial tissues sampled from AF patients.^35^ Take-off potential showed no significant difference between the groups (*Figure 5C*). Other hallmark AP parameters [mean diastolic potential (MDP), action potential amplitude (APA), maximal AP upstroke velocity (V_max_), plateau, and period] did not differ between paced and unpaced aEHTs (see Supplementary material online, *Figure S11*).

Western blots displayed a tendency towards lower protein levels of CASQ2 in paced aEHTs, which corresponds to findings in humans with AF,^36^ but the difference was not significant (see Supplementary material online, *Figures S12A* and *S13A*). Protein abundance or phosphorylation of CaMKII and p-CaMKII and phosphorylation of PKB (p-Akt) did not differ between groups (see Supplementary material online, *Figures S12B*–*D* and *S13B*–*D*).

Changes of the AP properties and structural remodelling, however, were clearly reflected in gene expression. The mRNA abundance of brain natriuretic peptide (NPPB) and actin alpha 1 (ACTA1) was higher in paced aEHTs (*Figure* *5E* and *J*). The mRNA abundance of the G protein-activated inward rectifier potassium channels 1 (KCNJ3, K_ir_3.1) and 4 (KCNJ5, K_ir_3.4), facilitating the acetylcholine-activated repolarizing current I_K,ACh_, was lower in the fast-paced group (*Figure* *5F* and *G*). The same held true for the potassium voltage-gated channel, shaker-related subfamily, member 5 (KCNA5, K_V_1.5), responsible for the ultra-rapid repolarizing current I_Kur_ (*Figure 5H*). Abundance of CASQ2 mRNA, on the other hand, was significantly higher (*Figure 5I*). These changes also corresponded with RNA-sequencing data (*Figure 5K–O*; Supplementary material online, *Figures S14* and *S15*) obtained in VChR1-transduced, intermittently long-term paced aEHTs from a pilot experiment, revealing robust, platform-overarching RNA expression results.

## Discussion

Persistent AF is complicated and perpetuated by remodelling. From a clinical perspective, force and coordination of contraction are reduced, facilitating thrombus formation and subsequent ischaemic events. The refractoriness is shortened, facilitating re-entry, probably contributing to high recurrence rate. Both findings could be replicated in goat and dog models of tachypacing.^37–39^ However, such large animal models are expensive and both technically demanding and ethically controversial. Genetic modification of large animals remains a challenge, complicating studies on the impact of gene variants associated with AF. Human iPSC-aCM can potentially overcome both limitations. They are comparably inexpensive, and patient-derived hiPSC-aCM allow for direct studies on gene variants. The results of the present study support the idea that tissue constructs from hiPSC-aCM may represent an attractive testing system in AF research.

### Continuous tachypacing serves to induce arrhythmia vulnerability in atrial engineered heart tissue

Early optogenetic tools used to tachypaced EHT showed a substantial desensitization within a few seconds.^14,40^ To allow for recovery from desensitization, pacing was performed only intermittently in the studies mentioned above. Interestingly, intermittent pacing using channelrhodopsin-2 as an optogenetic actuator was still sufficient to induce considerable electrical remodelling in vEHT. Three weeks of intermittent tachypacing (15 s tachypacing followed by 15 s of no pacing) rendered vEHT prone to induction of arrhythmias by burst stimulation.^14^ However, the same intervention was previously ineffective in aEHT.^41^ The failure to induce burst-evoked arrhythmias in aEHT was somewhat unexpected in this study, since both in humans and goats, shortening of refractoriness upon induced AF occurs within minutes or a few hours.^37^ This observation was also present *in vitro*, where in another recent 3D tissue model, this phenomenon occurred within 24 h.^34^ In human tissue slices, tachypacing at least acutely shortened APD.^42^ Our findings suggest higher resistance against tachypacing-induced remodelling in aEHT vs. vEHT. Our own previous findings and the published data, however, still suggested that tachypacing can induce AF-like electrical remodelling in aEHT. We deployed a tachypacing protocol that seemed more successful in longer-term tachypacing than most previous attempts.^34^ In our model, this protocol could only be achieved by careful selection of a suitable optogenetic actuator. For both PsCatCh2.0f and Chronos, faster opening kinetics than for CheRiff had been reported; additionally, Chronos displays faster closing kinetics.^22–24^ CheRiff had been previously used for cardiac research,^43–46^ and indeed, despite slower (but still fast, compared to e.g. ChR1) kinetic properties as judged by published properties, the improved version CheRiff2.0 proved to be best suitable for continuous tachypacing in our model. Thus, documented channel properties, such as excitation and emission wavelength and on- and off-kinetics, cannot replace testing of the actuator under the desired conditions. Many additional factors, such as membrane integrated fraction, channel protein half-life, ion selectivity, and desensitization, also play important roles. Apparently, the increased membranous expression and 5.5-fold larger photocurrents compared to CheRiff, which was achieved through optimization in this work, were the main contributing factors to its superiority to PsCatCh2.0f and Chronos in our hands.

### Continuous tachypacing induces contractile dysfunction within 24 h

In humans, several minutes of AF are sufficient to induce atrial contractile dysfunction that persists after cardioversion.^47^ Electrical and contractile remodelling during the first days of AF go hand in hand.^37^ As expected, the previous failure of intermittent tachypacing to induce shortening of APD was associated with a failure to induce contractile dysfunction.^41^ Conversely, aEHT continuous tachypacing for 24–72 h was needed to depress force, but still eventually successful (Seibertz *et al.*^34^ and this study; *Figure* *2B* and *C*). The comparably long duration needed to induce APD shortening might be attributable to hiPSC-aCM immaturity, a well-known limitation of the field. For specific questions, despite its very low availability, human atrial myocardial slices could represent an even more physiological *in vitro*/*ex vivo* testing system.^42,48,49^ For hiPSC-based models, this finding underscores the importance of longer-lasting, continuous tachypacing to replicate both AF-like aspects (electrical and contractile remodelling) in aEHT. Since in humans atrial contractile dysfunction can recover within several minutes after short episodes of rapid activation,^47^ our system could also be used to study reverse contractile remodelling and potential drug interventions, as aEHT contractile force similarly recovers after discontinuation of tachypacing (*Figure 2C*).

### Simple technical approach to study atrial fibrillation-like properties *in vitro*

To keep our model technically simple, it might have been preferable to develop an entirely LED-driven system. However, we anticipated that LED-based burst stimulation may not be feasible. Electrical stimuli at a fast rate (20 Hz) were suitable to induce a fibrillation-like state in aEHT. Optogenetic pacing at a similar frequency, however, would likely induce depolarization only, owing to the comparably slow off-kinetics of the respective channels. We had reasoned that at an APD_90_ of ∼200 ms (*Figure 5B*), fast flashing illumination would simply have summarized depolarization and would have been equivalent to constant illumination. Thus, we established a hybrid technique (LED for tachypacing and conventional field stimulation for burst application) consisting of two methods that are readily available and importantly can be performed without violation of sterility. All interventions can be carried out in the culture dish, with no necessity for a transfer to an organ bath as used for AP recordings. During transfer to the optical mapping setup, our model additionally proved robust in different media, such as FluoroBrite, and environments. Such an approach will also allow to study reverse remodelling in more depth upon cessation of tachypacing. More importantly, detection of arrhythmias is based on video-optical recording alone. Thus, our system may be more accessible to researchers without access to an AP recording setup and the corresponding expertise, though AP analysis obviously still provides valuable additional information on electrical remodelling of the tissue. The novel model allows the study of prolonged episodes of arrhythmia *in vitro* in a 3D model. It remains unclear how exactly fast alternating field stimulation induces arrhythmia. Nevertheless, detailed analysis of the consequences of optogenetic tachypacing clearly drew a connection to underlying alterations also occurring in AF.

In this regard, the physiological nature of the arrhythmia is of particular interest. Optical mapping analysis was somewhat hampered by the broad excitation range of the optogenetic actuator CheRiff2.0, which necessitated the use of strictly red excited dyes. Nevertheless, our data argued against macro-re-entry as the mechanism of arrhythmia. Re-entry would have moreover necessitated a combination of either long distances or slow conduction velocity with short refractoriness. Conduction velocity in EHT has been described around 160 mm/s,^29^ and the EHT length is around 8 mm at rod shape. In our aEHT, the effective refractory period is close to APD_90_, around 150 ms.^41^ Though the calculation may be over-simplifying, at an aEHT length of ∼8 mm, an AP would need (8 mm × 2)/160 mm/s = 100 ms to travel from one end to the other and back and thus less than APD_90_. As this represents the largest conduction distance, the calculation would not totally exclude re-entry, but render it unlikely. Consistent with these considerations, our mapping results suggest the induced activity of independent and uncoordinated centres of electrical activity as the potential mechanism underlying the fibrillation-like state. The interpretation of these results is, however, slightly limited, as with optical mapping, only APs and conduction near the surface of the tissue can be mapped and the broad excitation range of CheRiff2.0 required the use of a red-shifted voltage dye at sub-optimal excitation and emission spectra, leading to unfavourable signal-to-noise ratio. Termination of the tachyarrhythmia with short blue-light pulses demonstrated the acute reversibility of the BEEF phenomenon and resembled *in vivo* cardioversion therapy.

A likely contributing factor to the underlying arrhythmia mechanism is an alteration in cellular calcium handling. We observed a discordant CASQ2 expression (a non-significant trend towards lower protein expression accompanied by an increase in mRNA expression). The reason for this might be altered mature mRNA degradation due to miRNAs, which are known to reduce the expression of other major calcium handling proteins (e.g. L-type Ca^2+^ channel subunits and SERCA2).^50,51^ The increase in mRNA expression might be a subsequent effect compensating for an increase in mature mRNA and protein degradation.

### Triangulation of action potential shape and ion channel remodelling

In human AF, the shortening of APD_90_ associates with a prolongation of APD_20_, leading to a triangulated AP shape.^52^ This specific feature of AF could be replicated in our study (*Figure 5A–D*). The phenomenon relates to smaller transient outward currents in AF and is reflected in our assessment of ion channel expression (*Figure 5F–N*). Inward rectifier currents activated by muscarinic agents (I_K,ACh_; responsible subunits K_ir_3.1–3.4, encoded by *KCNJ3*, *5*, *6*, and *9*) are reduced in human persistent AF,^53,54^ a hallmark feature that could be replicated in our study (on an mRNA level), as well as in a similar *in vitro* model.^34^

The exact relevance of a triangulated AP for AF maintenance is not known. However, there is clear evidence that triangulation of AP shape has an impact on the response pattern to a block of the ultra-rapid potassium current, both in human atrium and in hiPSC-aCM.^8,55^ One underlying factor is a lower expression of the voltage-gated channel K_V1.5_, encoded by the *KCNA5* gene.^56–58^ This AF feature could again be replicated in our study and may become relevant when hiPSC-aCM will be used as a model for testing putative anti-AF drugs.^34^ Detection of triangulation as part of AF-like remodelling is impossible if controls are already triangulated.^34,41^ Thus, a sufficiently short APD_20_ (*Figure* *5A* and *D*) in control hiPSC-aCM, which resembles the situation in human sinus rhythm,^35^ seems to be a prerequisite for a useful application of hiPSC-aCM in the field of AF research.

### Summary and limitations

With our setup, we were able to induce episodes of tachyarrhythmia in aEHT (*Figure 3*). We demonstrated the key role of continuous optogenetic tachypacing to facilitate arrhythmia induction and present an adaptable way to achieve this. To our knowledge, successful induction of fibrillation-like episodes has not yet been shown in 3D engineered atrial tissue. Furthermore, several hallmark features of AF could be successfully recapitulated in our model and may have helped to render the induction successful. These included a reversible decline of contractility, including an uncoordinated contraction phenotype (‘wobbly’), triangulation of AP shape, typical changes in gene expression, and alteration of structural tissue integrity. Tachyarrhythmia could successfully be terminated by constant illumination with blue light and subsequent depolarization block, and mapping experiments suggested multiple foci of automaticity as the main mechanism for our *in vitro* model of AF.

The comparability of our model to *in vivo* AF, however, is of course somewhat limited. We used hiPSC-aCM only; the tissue did not contain any other specific cell types. Since fibrosis and inflammation are among the most prominent pathophysiological mechanisms in AF, the absence of fibroblasts and immune cells, in particular, is a limitation, which will be addressed in the future. Another limitation is the immaturity of the hiPSC-aCM, especially with regard to spontaneous contraction, which is in contrast to native human atrial cardiomyocytes. This limitation affects hiPSC-derived tissue models in general. Despite this, our model was able to recapitulate AF-like features and electrophysiological remodelling with sufficient accuracy to allow for continuous and reliable induction of arrhythmia. This underscores how our model reflects *in vivo* AF pathophysiology to a degree potentially exceeding the possibilities of previous *in vitro* models. Lastly, optical mapping suffered from poor signal-to-noise ratio, which resulted in qualitative rather than quantitative mapping data. We used the potentiometric dye RH237 for our optical mapping experiments. Due to the green-shifted spectrum of eYFP attached to the CheRiff2.0 channel protein (excitation peak ∼514 nm, emission peak ∼529 nm) and the broad excitation range of CheRiff2.0, we had to use a red-shifted light source (635 nm) for excitation of the RH237 dye. The considerable spectral distance from its optimal excitation spectrum resulted in suboptimal signal-to-noise-ratio. In aEHTs without spectral limitations, caused by CheRiff2.0 and eYPF expression, we provided evidence for good overall performance of RH237 and our optical mapping setup, but in CheRiff2.0 aEHT, numerical AP analysis was precluded. The interpretation of optical mapping results may be additionally compromised by an unclear extent of tissue penetration of the potentiometric dye. However, our aEHT method follows highly standardized protocols yielding predictable cell distribution with concentration on the tissue surface,^59^ in turn leading to predominant contribution of the near-surface cardiomyocytes to the signal.

We therefore strongly believe that in spite of its limitations, our approach of optogenetic tachypacing to induce arrhythmia in aEHT is an important milestone in the study of AF and its molecular and cellular pathophysiology. Arrhythmic potential can easily be quantified via percentage of aEHTs showing sustained tachyarrhythmia after repeated induction attempts. In future studies, we aim to use our findings for the assessment of putative pro- or anti-arrhythmic interventions.

## Supplementary Material

euag158_Supplementary_Data

## Data Availability

Most data are displayed as individual data points from individual replicates and are thus incorporated directly in the article and its supplement. Code for the pacing device is available online at https://github.com/owlcap0ne/pacer_led_board. Additional raw data underlying this article will be shared on reasonable request to the corresponding author. Sequence data on the improved channelrhodopsin are subject to an embargo of 12 months from the publication date of the article. Once the embargo expires, the data will be available upon reasonable request from the corresponding author respecting possible patent circumstances.
