# Supplementary material for: Optogenetic tachypacing facilitates burst-induced arrhythmia in stem cell-derived human atrial engineered heart tissue
Source: Europace. 2026 Aug 18;28(8):euag158. doi: 10.1093/europace/euag158 (PMC13484016; doi:10.1093/europace/euag158)
Supplement: Supplementary file 1 [file euag158_supplementary_data.zip › Supplementary_figures.pdf]

Supplement

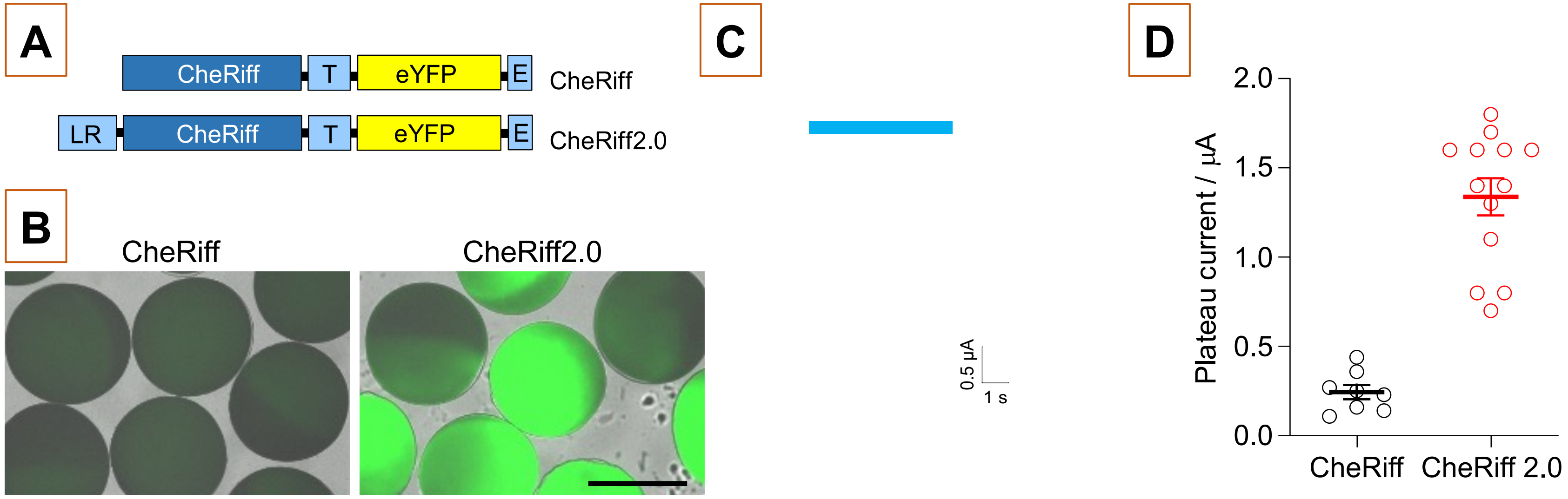

CheRiff	CheRiff2.0
0.27	0.7
0.25	0.8
0.14	1.7
0.11	1.6
0.23	1.4
0.16	1.6
0.36	0.8
0.44	1.3
	1.4
	1.6
	1.1
	1.8
	1.6

Supp. Fig. S1. Generation of CheRiff2.0 with enhanced expression and photocurrent in *Xenopus laevis* oocytes. A: Schematic diagram of CheRiff and CheRiff2.0. LR: cleavable N-terminal signal peptide Lucy–Rho; T: plasma membrane trafficking signal from Kir2.1; E: endoplasmic reticulum export signal from Kir2.1. **B:** Fluorescence images (Leica DMi8 microscope) of *Xenopus laevis* oocytes expressing CheRiff and CheRiff2.0. 20 ng cRNAs injected for each, scale bar = 1 mm. **C:** Representative photocurrent traces of CheRiff (black) and CheRiff2.0 (red). Blue bar indicates 5 s blue light illumination. **D:** Photocurrents of CheRiff and CheRiff2.0. CheRiff, n = 8; CheRiff 2.0, n = 13; Error bars = SEM, data chart on right. For C and D, illumination was performed with a 473-nm laser at an intensity of 1 mW/mm². Holding potential was kept at −60 mV. Measurements were performed with a bath solution containing 110 mM NaCl, 2 mM $BaCl_2$, pH 7.6.

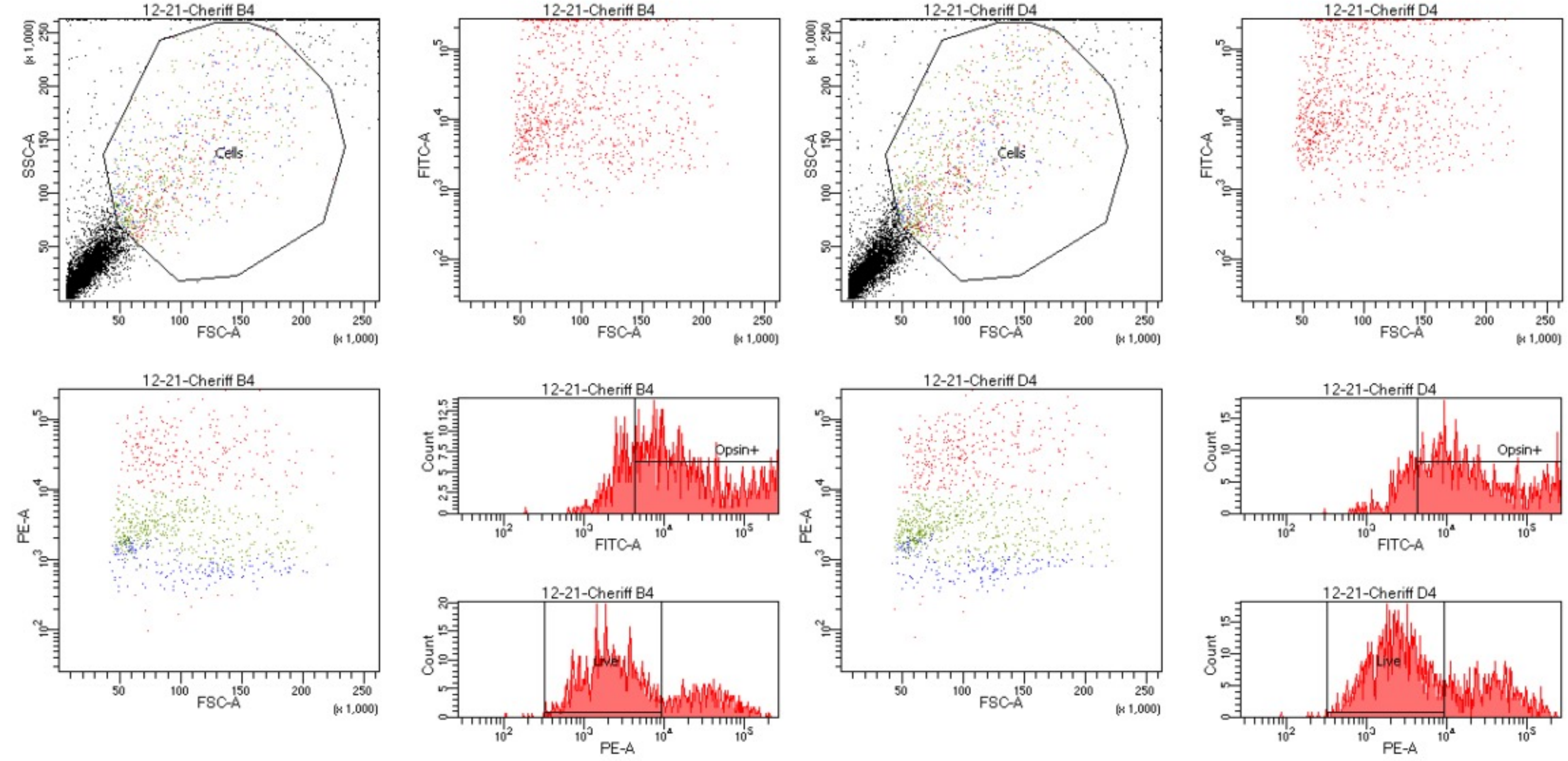

Tube: Cheriff B4

Population	#Events	%Parent	%Total
All Events	10,000	####	100.0
Cells	903	9.0	9.0
Live	636	70.4	6.4
Opsin+	457	71.9	4.6

Tube: Cheriff D4

Population	#Events	%Parent	%Total
All Events	10,000	####	100.0
Cells	1,055	10.5	10.5
Live	706	66.9	7.1
Opsin+	535	75.8	5.4

Supp. Fig. S2: Titration of virus multiplicity of infection (MOI). Several consecutive experiments were performed in order to determine an MOI that allows for 70-90% of YFP+ cells, *i.e.* opsin-expressing cells. The example analysis displays two different EHTs (B4 and D4) with a percentage of opsin-expressing cells (here: CheRiff2.0) of 71.9 and 75.8, respectively. YFP quantified via FITC settings, PE-A channel used for viability stain, gating strategy in bottom panels.

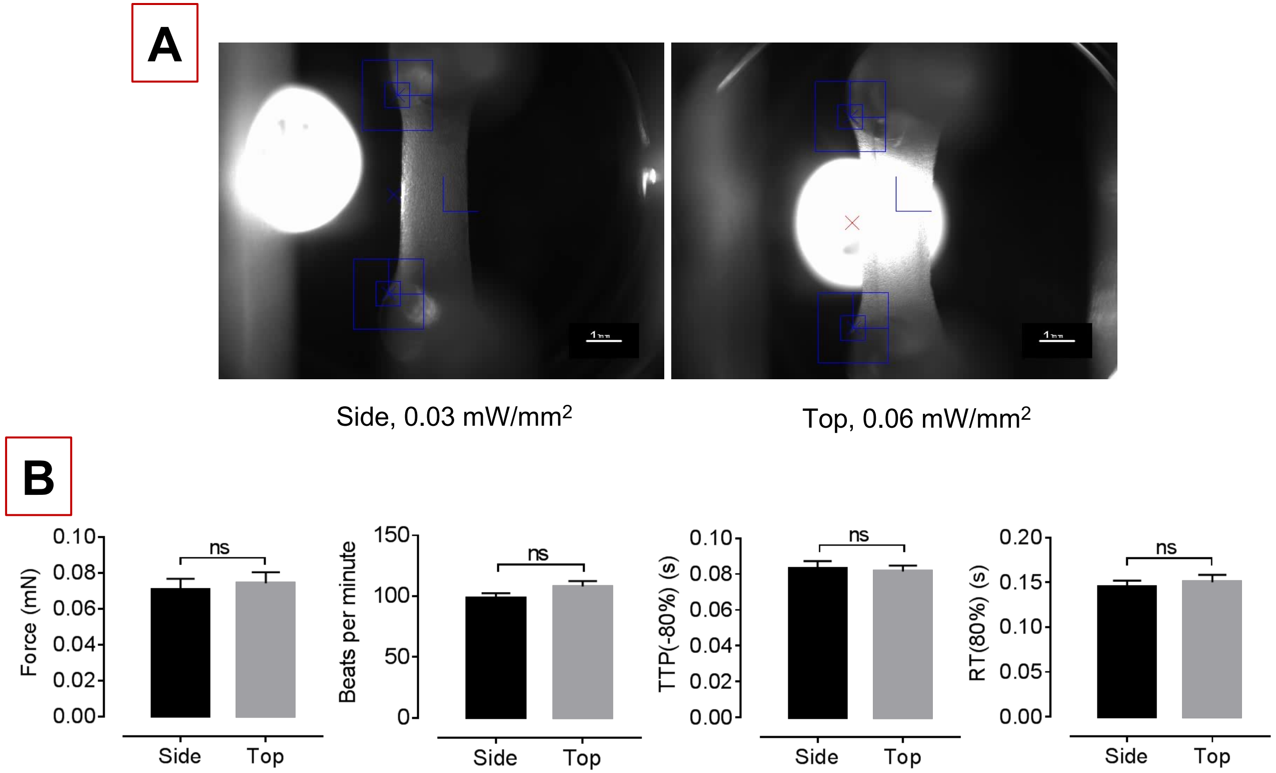

Supp. Fig. S3: Evaluation of the influence of red light on VChR1-aEHTs[30]. A: Left: aEHTs were placed towards the side of the LEDs (Side group) and exposed to red light at 0.03 mW/mm^2. Right: aEHTs were placed directly on top of LEDs (Top group) and exposed to red light at 0.06 mW/mm^2. Scale bar 1 mm. **B:** Contractility parameters (force, BPM, TTP and RT at 80%) of VChR1 RA-EHTs were compared between Side and Top groups (n = 11 EHTs per group). Data are presented as mean ± SEM. ns: not significantly different by unpaired t-test.

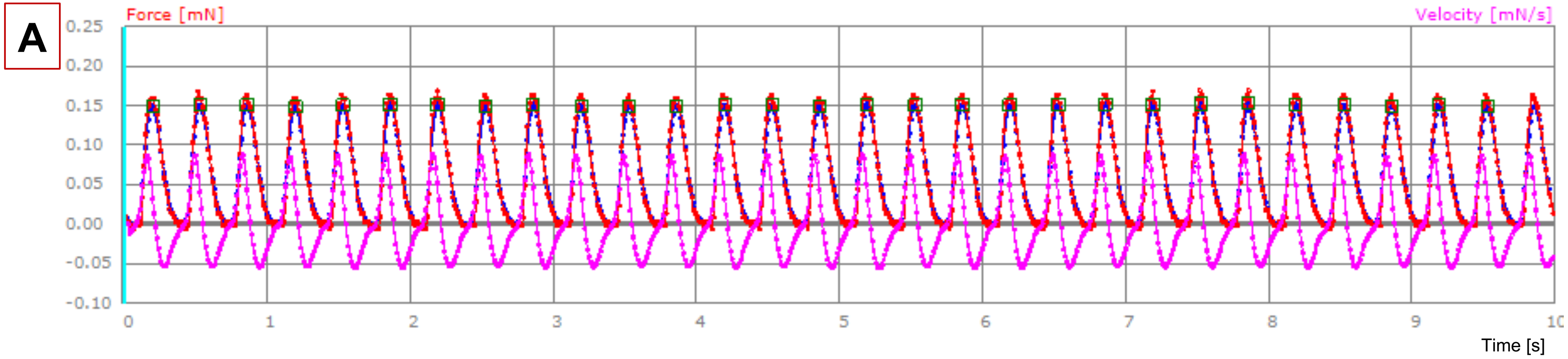

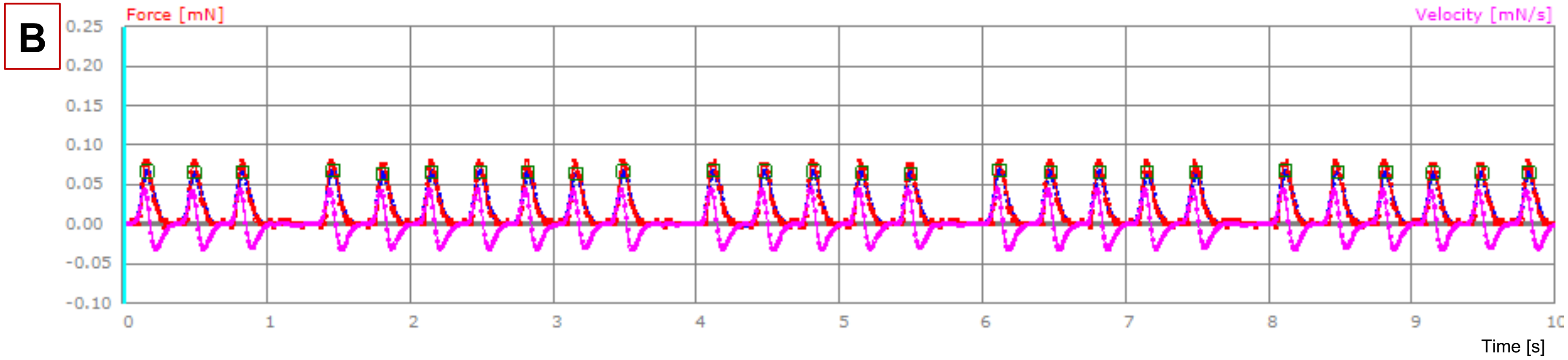

Supp. Fig. S4: Representative original recording traces of optically paced CheRiff2.0-aEHT. A: Example of aEHT with 100% optical pacing adherence at 3.0 Hz, pulse duration 40 ms. **B:** Atrial EHT with partial loss of capture every 3-4 beats, resulting in an arrhythmic beating pattern. Red traces and peaks represent contraction, pink traces represent contraction velocity, green squares indicate peak recognition by software.

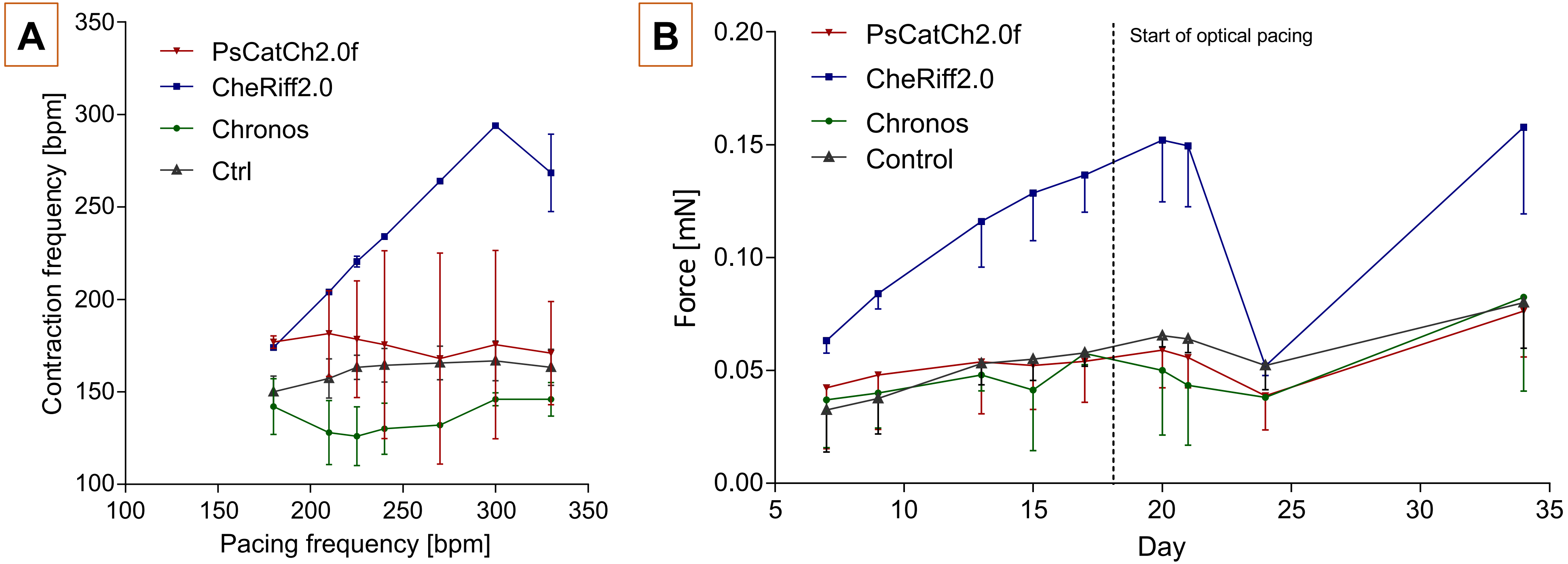

Supp. Fig. S5: Pacing adherence and contractility depending on choice of optogenetic actuator. A: Pacing adherence. Horizontal axis displays the optogenetic pacing frequency of the LEDs, vertical axis displays the atual contraction frequency of the aEHTs transduced with the respective optogenetic actuator (PsCatCh2.0f, CheRiff2.0, or Chronos). Pulse duration was 40 ms. Transduction rate was 70-90% for all three actuators tested. Only aEHTs expressing CheRiff2.0 were able to follow optogenetic pacing reliably. N = 3–5 aEHTs per group. **B:** Force development of aEHTs expressing different optogenetic actuators over time. AEHTs expressing CheRiff2.0 developed a contraction force more than twice as high as those expressing other actuators or non-transduced control aEHTs. After pacing onset, force declined drastically, but recovered after the end of the pacing period. N = 3–5 aEHTs per group.

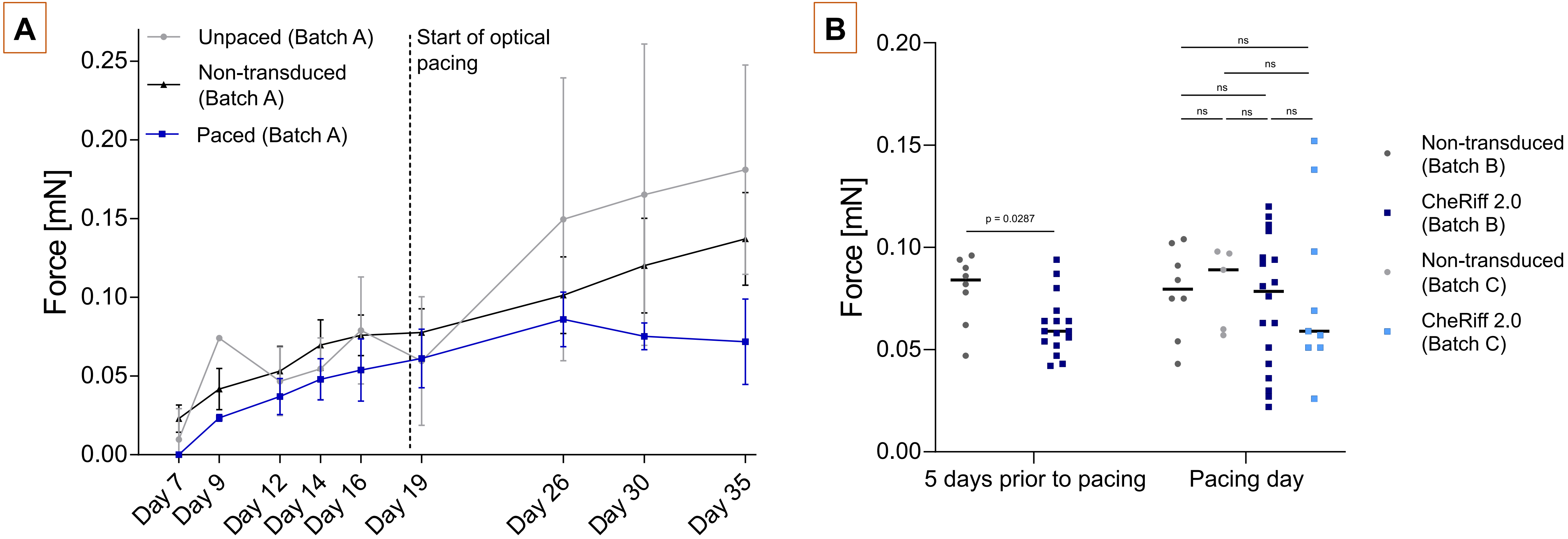

Supp. Fig. S6: Force development over time in transduced and non-transduced aEHTs. A: AEHT batch A force development. Difference in force between paced and unpaced aEHTs was significant on day 35 only (end of pacing period). Comparison of unpaced aEHTs and non-transduced aEHTs was significant at no time point. N = 4-7 aEHTs per group, unpaired Welch's t test. **B:** Force prior to pacing in additional batches B and C. Five days prior to pacing, force of non-transduced (NT) aEHTs was higher than in CheRiff 2.0-transduced aEHTs. Immediately prior to commencing the pacing period, force did not differ between groups. N = 5-18 aEHTs per group, unpaired Welch's t-test.

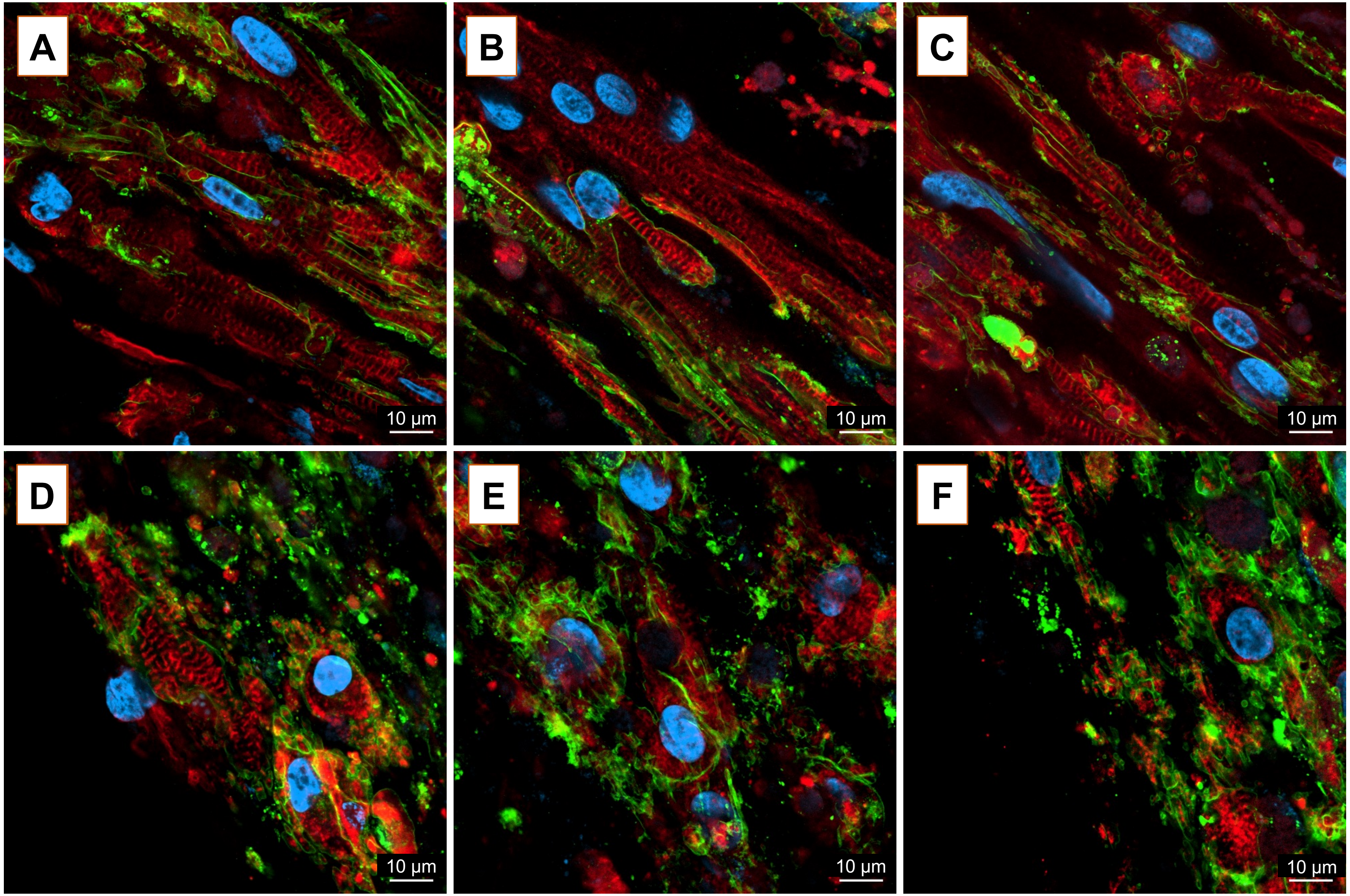

Supp. Fig. S7: Confocal immunofluorescence images of aEHTs after pacing period. DAPI (blue), eYFP (green), and alpha-actinin (red). **A-C:** Representative images of non-paced aEHTs. **D-F:** Representative images of optically tachypaced aEHTs. Sarcomeric disarray can be observed in paced aEHTs.

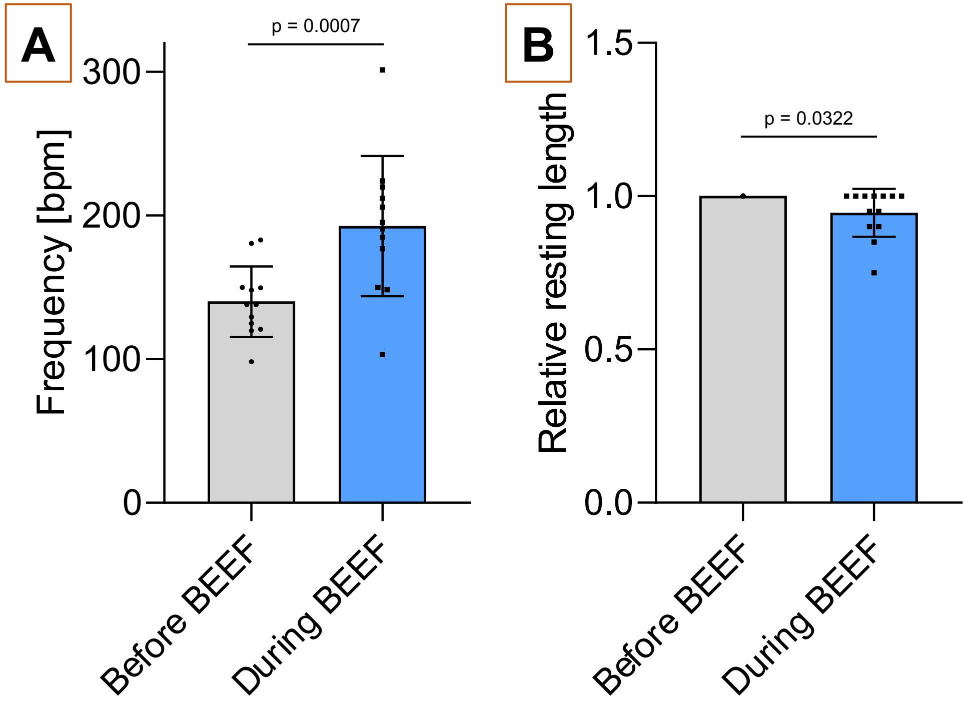

Supp. Fig. S8: Contraction parameters before and during BEEF. A: Contraction frequency of aEHTs right before and during BEEF. N = 12 aEHTs from 5 batches. Paired t test. **B:** Relative resting length of aEHTs right before and during BEEF. N = 13 aEHTs from 5 batches. Ratio paired t-test.

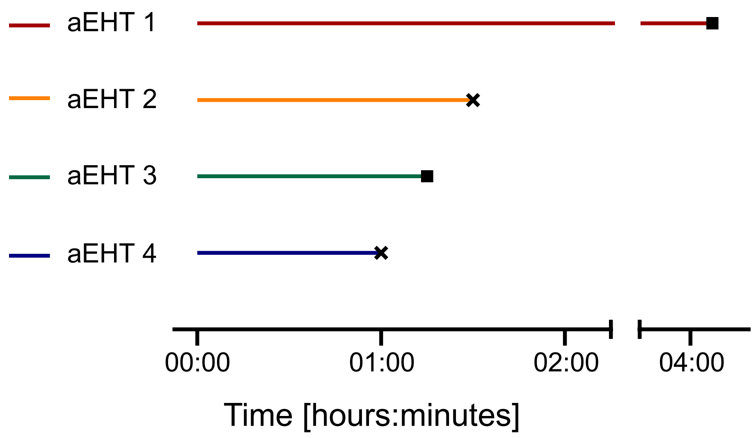

Fig. S9: BEEF duration. In 4 aEHTs showing persisting BEEF, arrhythmia duration was assessed systematically. In this persisting („type 2") BEEF phenotype, arrhythmia did not terminate spontaneously. Measurements were discontinued only if contraction force was too weak for the analysis software to track (box), or when BEEF was terminated by applying a short blue light pulse (x). Blue light pulse duration sufficient to terminate arrhythmia was 40 and 10 ms for aEHTs 2 and 4, respectively.

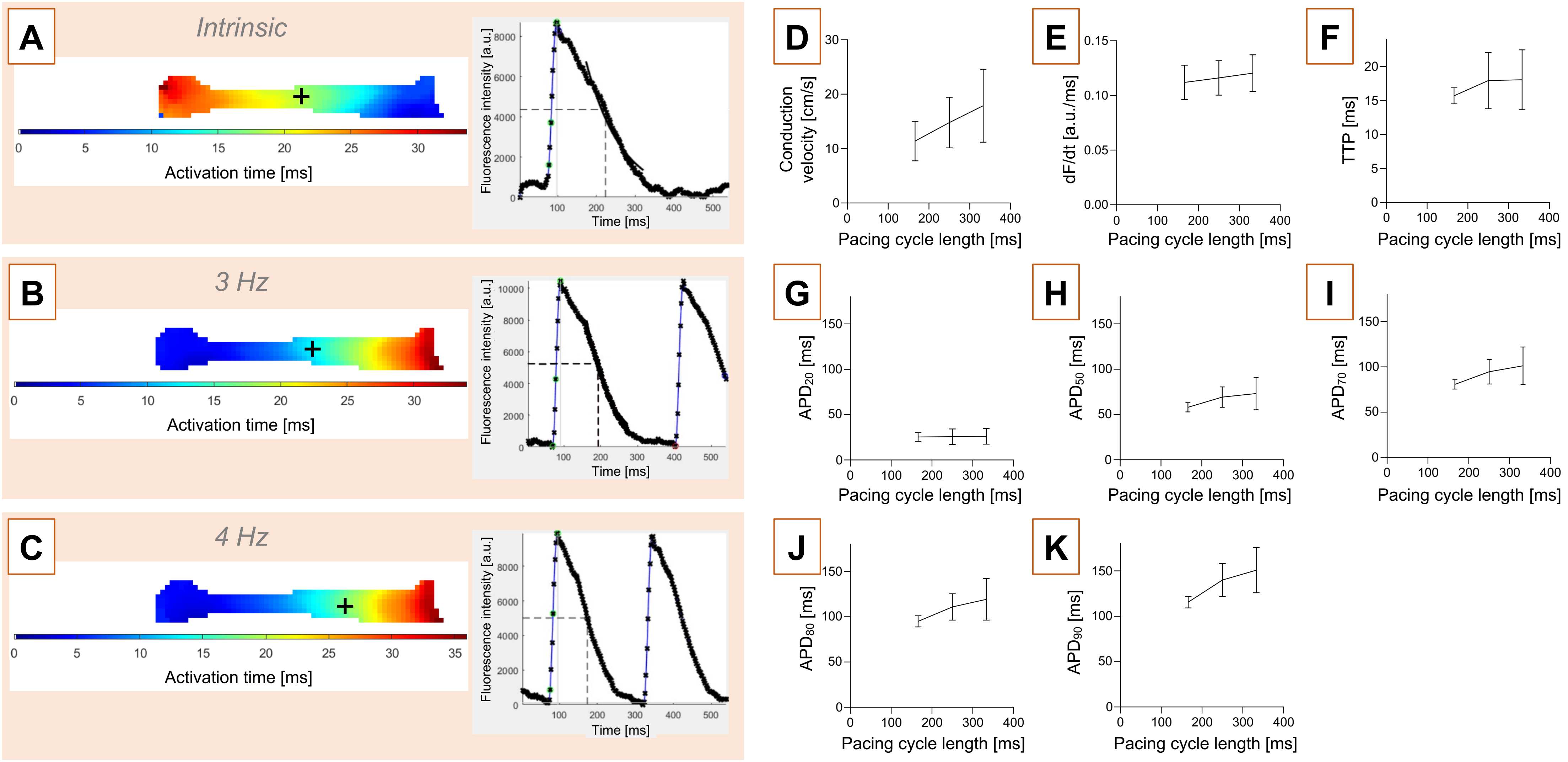

Supp. Fig. S10: Optical voltage mapping with RH237 at 550 nm illumination. A–C: Maps showing the propagation of activation in a representative aEHT. Color codes for the time at which depolarization midpoint was reached for each pixel. Representative single-pixel action potentials with location of the pixel within the map indicated below the AP. Data are averaged over at least 50 contraction cycles, depending on frequency, **A:** for intrinsic activation, **B:** for electrical pacing at 3 Hz, and **C:** for electrical pacing at 4 Hz. **D–K:** Graphs showing restitution curves at different pacing cycle lengths (166 ms ≙ 6 Hz, 250 ms ≙ 4 Hz, 333 ms ≙ 3 Hz), **D:** for conduction velocity, **E:** for mean upstroke velocity or dF/dt in arbitrary units per millisecond [a.u./ms], **F:** for time to peak, **G:** for action potential duration at 20% repolarization (APD_{20}), **H:** for APD_{50}, **I:** for APD_{70}, **J:** for APD_{80}, **K:** for APD_{90}. Error bars represent standard deviation. N = 4.

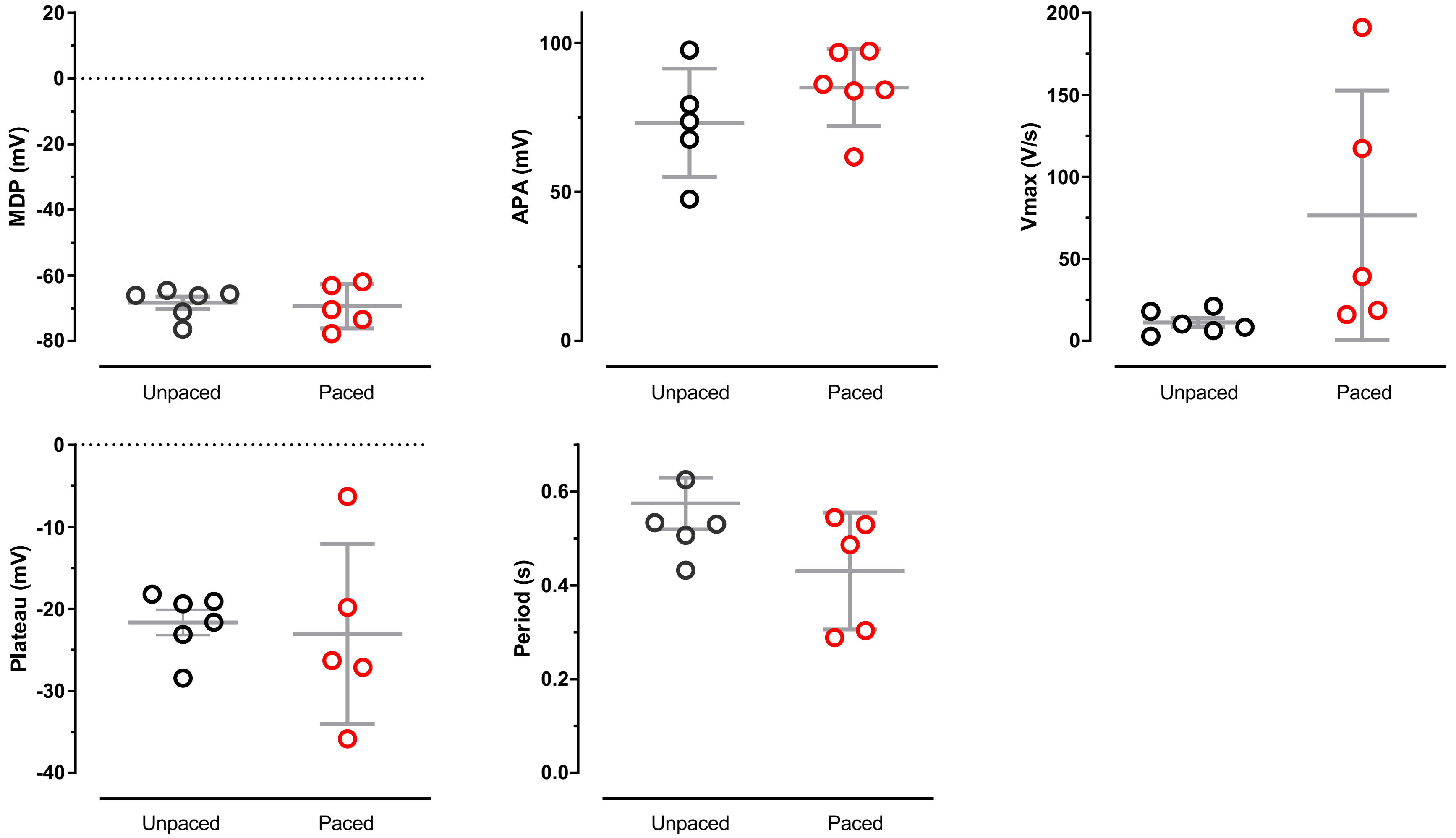

Supp. Fig. S11: Additional action potential parameters of paced vs. unpaced aEHTs. No significant differences. MDP = mean diastolic potential, APA = action potential amplitude, Vmax = maximal AP upstroke velocity, plateau = plateau phase voltage, period = period between AP peaks; n = 5-6 aEHTs per group from 2 batches, unpaired t-test with Welch's correction.

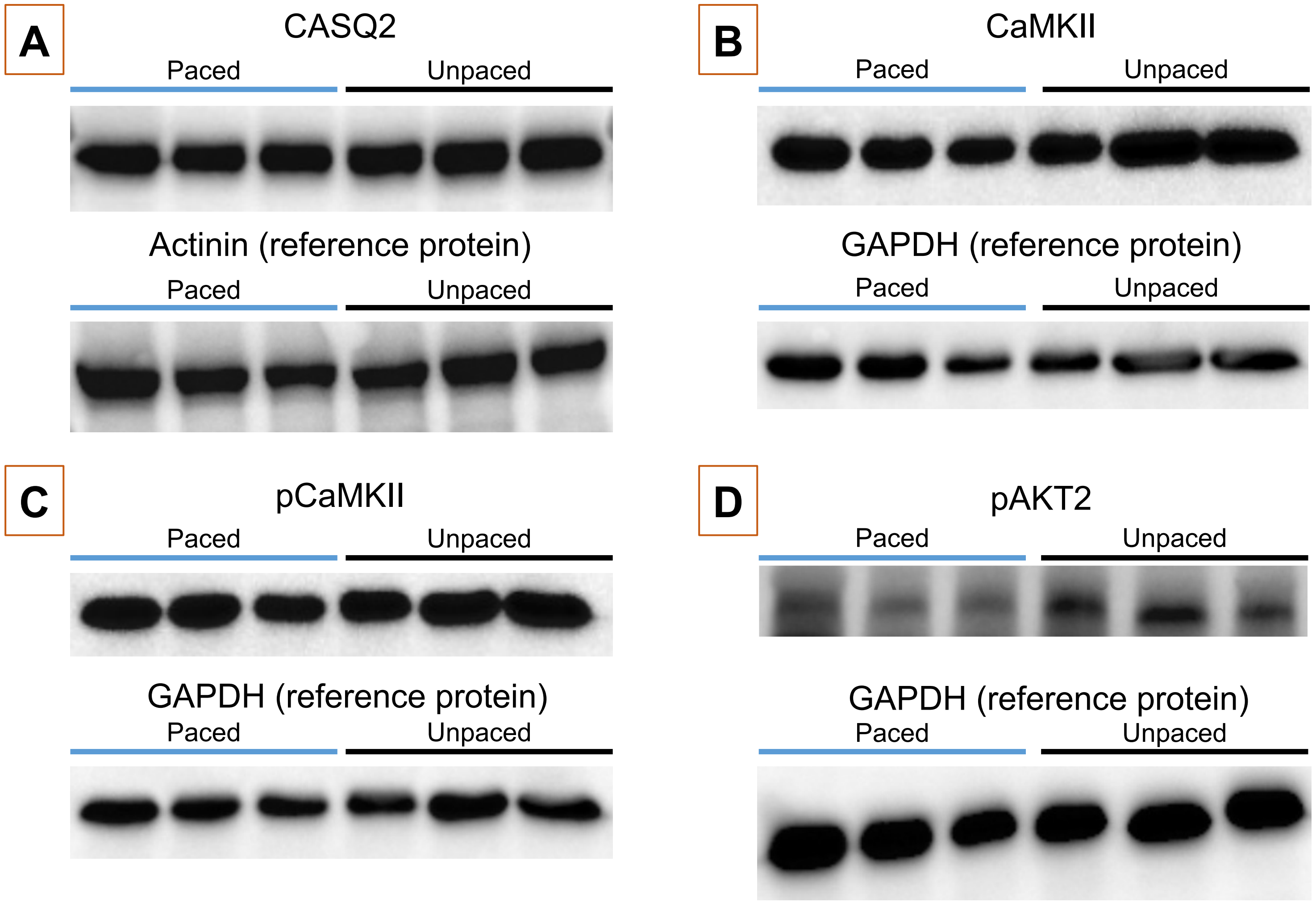

Supp. Fig. S12: Original Western blots. A: Calsequestrin 2 (CASQ2). **B:** Ca^{2+}/calmodulin-dependent protein kinase II (CaMKII). **C:** Phosphorylated CaMKII, Thr286. **D:** Phosphorylated protein kinase B, isoform 2 (pAKT2). Respective loading control (reference protein) shown below respective blot.

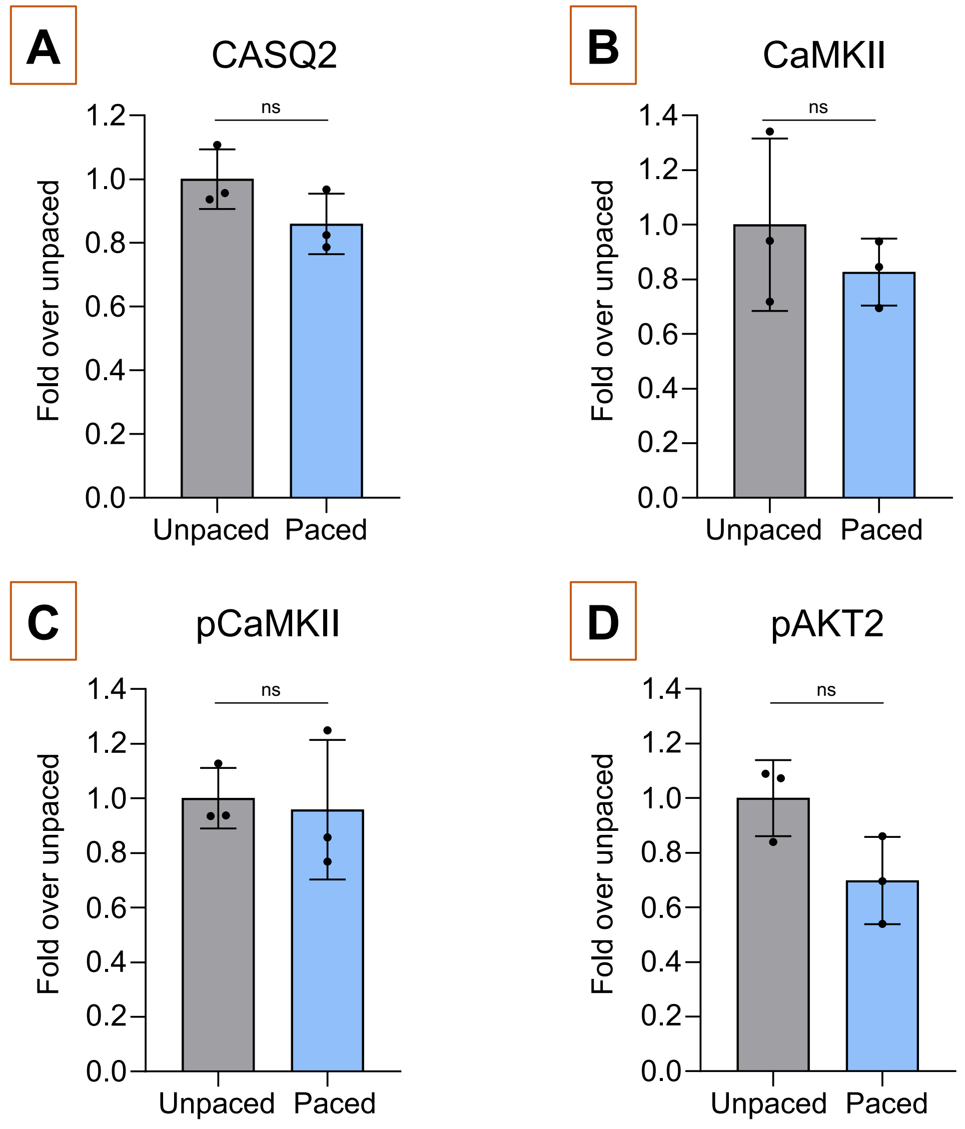

Supp. Fig. S13: Abundance of selected proteins analyzed via Western blot in paced aEHTs and unpaced controls. A: CASQ2 (Calsequestrin 2). **B:** CaMKII (Ca^{2+}/calmodulin-dependent protein kinase II). **C:** pCaMKII (Phosphorylated CaMKII, Thr286). **D:** pAKT2 (Phosphorylated protein kinase B, isoform 2). N = 3 aEHTs per group. Unpaired t-test with Welch‘s correction, no significant differences.

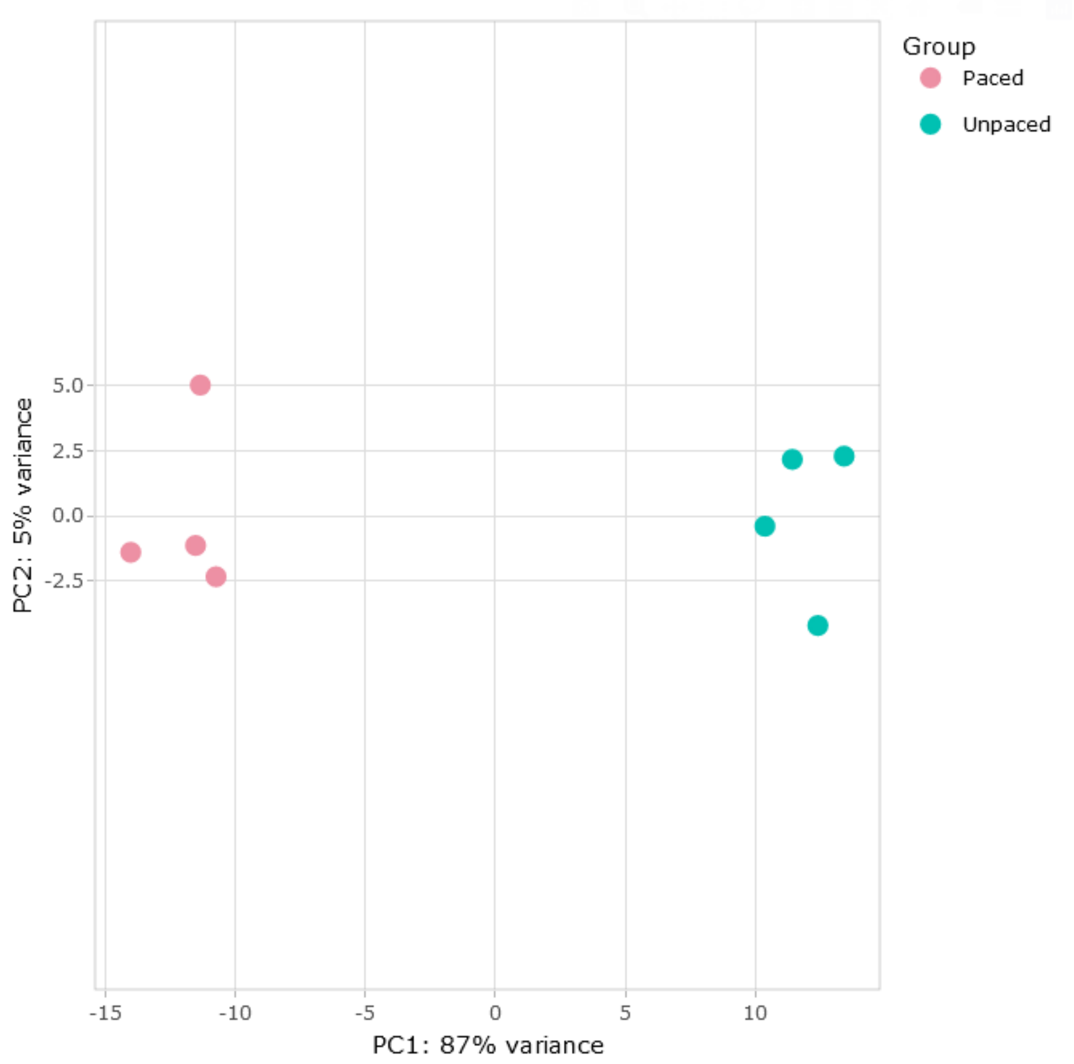

Supp. Fig. S14: Principal component analysis (PCA) of RNAseq data of aEHT expressing VChR1, intermittently fast-paced for 21 days. The intervention „paced vs. unpaced“ generated a clear clustering, 87% of variance could be explained by the intervention.

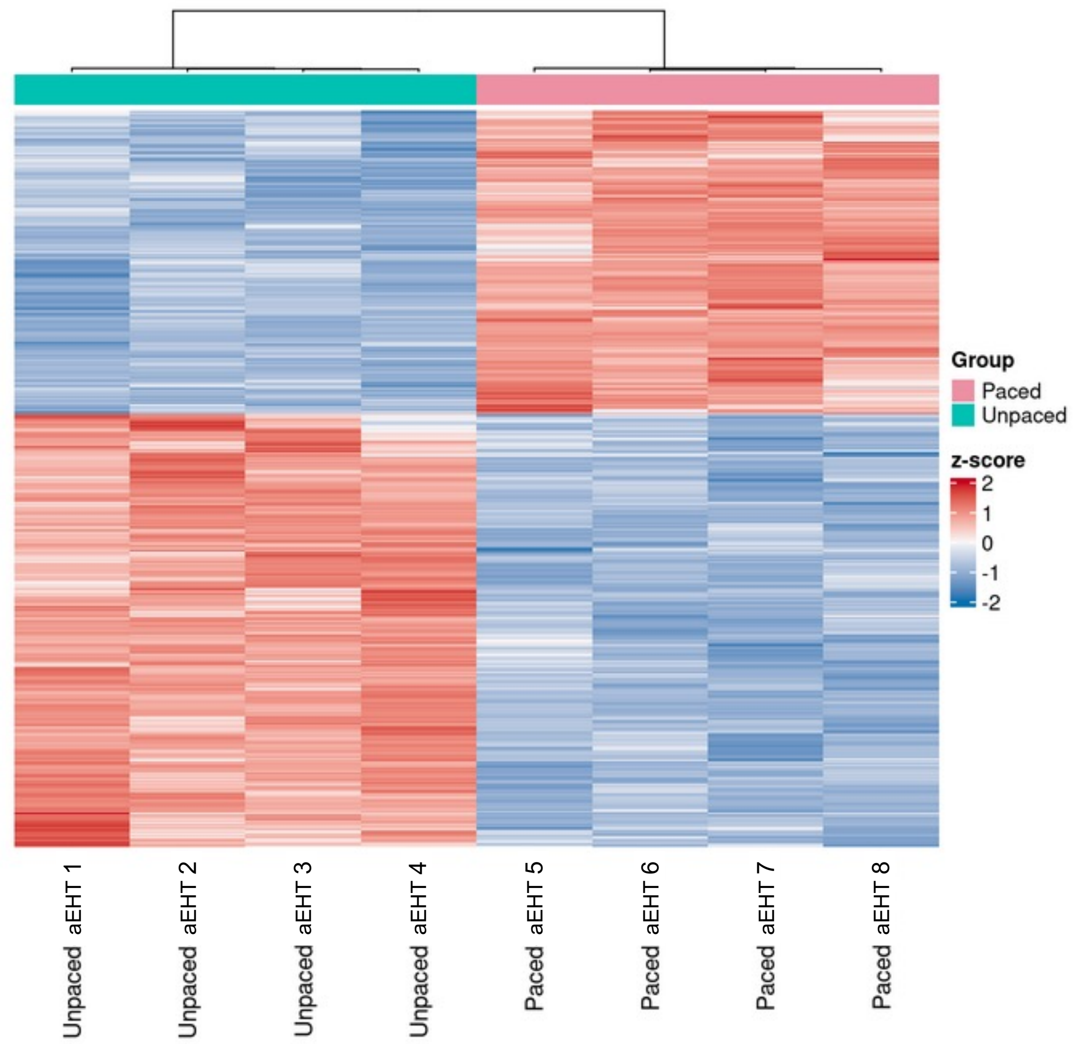

Supp. Fig. S15: Heatmap and cluster analysis of RNAseq data of aEHT expressing VChR1, intermittently fast-paced for 21 days. Hierarchical clustering shows clear separation of paced vs. unpaced aEHTs according to groups.
