# Supplementary material for: Optogenetic tachypacing facilitates burst-induced arrhythmia in stem cell-derived human atrial engineered heart tissue
Source: Europace. 2026 Aug 18;28(8):euag158. doi: 10.1093/europace/euag158 (PMC13484016; doi:10.1093/europace/euag158)
Supplement: Supplementary file 1 [file euag158_supplementary_data.zip › Supplementary_table_T1.pdf]

Supplementary table T1: Primer pairs for qPCR

Gene	Sequence
NPPB	TGCAAGGGTCTGGCTGCTTTGG
	CACTTCAAAGGCGGCCACAGGG
ACTA1	AGCAGTTGTAGCTACCCGCCCA
	GACCATGACGCCCTGGTGTCG
KCNJ3	AAAAACGATGACCCCAAAGA
	TGTCGTCATCCTAGAAGGCA
KCNJ5	GTGAAGGGACAGGATGGCTTA
	GCTGGGATGTTGTTGAGATCG
KCNA5	CGAGGATGAGGGCTTCATTA
	CTGAACTCAGGCAGGGTCTC
CASQ2	AGCAGCTGAACACTTCCAGCCT
	AGGCGACGTAGAGTGGGTCTTT
